# Trace Elements in the Pancreas: From Physiological Homeostasis to the Pathogenesis of Diabetes, Pancreatitis, and Cancer—A Review

**DOI:** 10.3390/life16050864

**Published:** 2026-05-21

**Authors:** Łukasz Bryliński, Katarzyna Brylińska, Jolanta Sado, Kacper Kraśnik, Miłosz Smyk, Olga Komar, Filip Woliński, Alicja Forma, Katarzyna Rusek, Jolanta Flieger, Grzegorz Teresiński, Jacek Baj

**Affiliations:** 1 Department of Forensic Medicine, Medical University of Lublin, ul. Jaczewskiego 8b, 20-090 Lublin, Poland; lukbry2@gmail.com (Ł.B.); katarzynarusek02@gmail.com (K.R.); jacek.baj@umlub.edu.pl (J.B.); 2 Doctoral School, Medical University of Lublin, ul. Chodźki 7, 20-093 Lublin, Poland; 3 Department of Correct, Clinical and Imaging Anatomy, Medical University of Lublin, ul. Jaczewskiego 4, 20-090 Lublin, Poland; 4 Student Scientific Society of Correct, Clinical and Imaging Anatomy, Medical University of Lublin, ul. Jaczewskiego 4, 20-090 Lublin, Polandmiloszs.105@gmail.com (M.S.); olgakomar720@gmail.com (O.K.); 5 Student Scientific Society of Forensic Medicine, Medical University of Lublin, ul. Jaczewskiego 8b, 20-090 Lublin, Poland; jolanta.flieger@umlub.edu.pl; 6 Department of Analytical Chemistry, Medical University of Lublin, ul. Chodźki 4a, 20-093 Lublin, Poland; grzegorz.teresinski@umlub.edu.pl

**Keywords:** pancreas, pancreatitis, diabetes, pancreatic cancer, element, metallomics, elemental concentration, toxic element, heavy metal, micronutrients

## Abstract

The pancreas is an organ with two functions: endocrine and exocrine. The proper functioning of the pancreas depends on many factors. One of these is trace elements—precise control of trace element homeostasis is important for both the endocrine and exocrine parts. This review provides a comprehensive summary of current knowledge regarding the role of trace elements: iron (Fe), copper (Cu), cobalt (Co), iodine (I), manganese (Mn), zinc (Zn), silver (Ag), cadmium (Cd), mercury (Hg), lead (Pb), and selenium (Se) in pancreatic physiology and their influence on the pathogenesis of key diseases of this organ, such as diabetes (DM), acute (AP) and chronic pancreatitis (CP), autoimmune pancreatitis (AIP), and pancreatic cancer (PC). Trace elements, including Fe, Cu, Zn, Se, and Mn, play a fundamental role in maintaining endocrine and exocrine homeostasis, participating in insulin synthesis, stabilizing digestive enzymes, and the functioning of antioxidant systems. It has been demonstrated that disturbances in their concentrations lead to the activation of pathological molecular pathways, including oxidative stress, chronic inflammation, and beta-cell apoptosis. In the context of diabetes, excess Fe promotes ferroptosis, whilst exposure to heavy metals such as Cd, Pb, and Hg induces insulin resistance and pancreatic islet dysfunction. In the course of pancreatitis, elements such as Zn and Se exhibit protective potential by stabilizing tissue barriers, whereas toxic metals impair ion transport, exacerbating fibrotic processes. Furthermore, analysis of available data indicates a significant association between heavy metal accumulation and pancreatic carcinogenesis, driven by DNA damage and oncogene modulation. Understanding pancreatic metallomics opens new prospects for early diagnosis, environmental prevention, and the development of targeted therapeutic strategies that restore the body’s micronutrient balance.

## 1. Introduction

The basic building blocks of all living matter are elements. Of all the elements found in nature, about 50 occur in living organisms in measurable concentrations. In this group, we can distinguish trace elements: those for which the body’s requirement is approximately 100 mg per day [1]. They play a critical role in the functioning of the body: they are cofactors of various enzymes and antioxidant molecules that enable the body to function properly [2], Zinc (Zn) plays a cofactor role in nearly 300 different enzymes, and copper (Cu) and Zn are the building blocks for Cu-Zn superoxide dismutase (Cu/Zn-SOD), while selenium (Se) is a component of glutathione peroxidase (GPx) [3,4,5]. In addition, trace elements also participate in the stabilisation of protein structures and mediate signalling pathways [6,7]. Despite their numerous functions, trace elements at inappropriate concentrations can harm the body by disrupting cellular redox balance and leading to numerous pathologies, which is why their impact on human functioning is twofold [8].

Being essential for the proper functioning of cells, trace elements also affect the endocrine system and hormone synthesis—proper levels of Se, Zn, Cu, iron (Fe), and manganese (Mn) are essential for the proper functioning of the thyroid gland, and iodine (I) is a component of the hormones produced by the thyroid gland [9]. Exposure to lead (Pb) may affect the production of follicle-stimulating hormone (FSH) in women [10]. Adequate concentrations of trace elements are also required for normal ovarian function and sex hormone synthesis [11]. Trace elements also affect the testes: their accumulation in testicular tissue leads to a decrease in testosterone, a reduction in sperm quality, and may also cause testicular inflammation. Cadmium (Cd) may induce Leydig cell apoptosis [12,13]. Exposure to Cd can affect the production of steroid hormones in the adrenal glands leading to increased corticosteroid synthesis [14].

**Figure 1:**
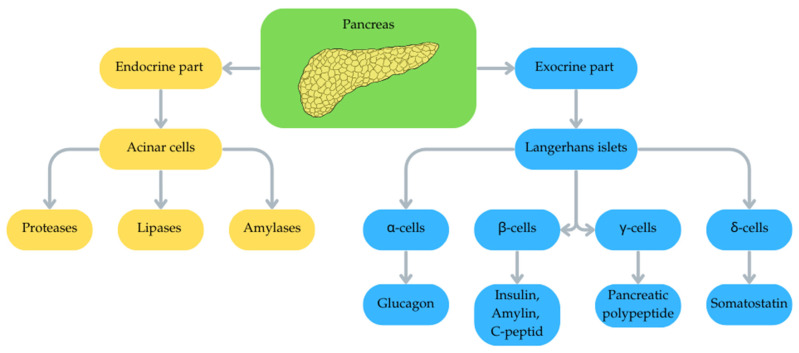
The structure of the pancreas and the synthesis of hormones and digestive enzymes by individual cells.

The pancreas is an organ with a dual nature: located in the retroperitoneal space, behind the stomach, in proximity to the duodenum, liver and spleen, it performs both exocrine functions, producing and secreting digestive enzymes such as pancreatic proteases (3 forms of trypsin; chymotrypsinogen A and B; proelastase, procarboxypeptidase), pancreatic lipases and amylases, as well as endocrine functions, producing hormones responsible for regulating blood glucose levels and visceral secretion [15,16]. The endocrine part is composed of Langerhans islets, whose architecture is based on five main types of endocrine cells. The most numerous population within the islets is β-cells, which account for 65% to 80% of the total number of cells. Their function is to produce and secrete insulin, a peptide with a hypoglycaemic effect (i.e., lowering blood glucose levels), as well as amylin and C-peptide. α-cells are the second-largest fraction, comprising 15% to 20% of the islet cell population. These cells are responsible for secreting glucagon, an insulin antagonist hormone that raises blood glucose levels. δ-cells constitute 3% to 10% of islet cells and produce somatostatin, which acts paracrinally, regulating the release of both glucagon from α-cells and insulin from β-cells. γ-cells, also known as pancreatic polypeptide cells, account for 3% to 5% of endocrine cells. The PP they secrete has a regulatory function, affecting both exocrine and endocrine pancreatic secretory activity. The smallest population is represented by epsilon cells, which account for less than 1% of the total islet cell population. These cells are the source of the hormone ghrelin [17]. Many factors can influence pancreatic function and the development of pathologies: genetic (genetic predisposition and gene mutations), environmental and metabolic (poor diet, excessive alcohol consumption, obesity). These also include trace elements (Figure 1) [18,19].

This review aims to gather information on the role of trace elements: Fe, Cu, cobalt (Co), I, Mn, Zn, silver (Ag), Cd, mercury (Hg), Pb, and Se in the functioning of pancreatic cells in the context of both exocrine and endocrine cells. The article presents, in an innovative and structured manner, the impact of these elements on pancreatic diseases: diabetes mellitus (DM), acute (AP) and chronic pancreatitis (CP), autoimmune pancreatitis (AIP), and pancreatic cancer (PC). We focus on the impact of these trace elements on the pathogenesis, development, and course of diseases, discussing their effects at the level of individual cell types and molecular pathways. Furthermore, we discuss their potential application in treatment. Based on the current state of knowledge, we hypothesise that the progression from pancreatic metabolic disorders to chronic inflammation and malignant tumours is driven by metal-dependent disturbances: the breakdown of Se- and Zn-based defence mechanisms, the activation of cell death via Fe and Cu (ferroptosis/cuproptosis) and the accumulation of pro-inflammatory heavy metals (Cd, Hg, Pb). This concept suggests that restoring trace element homeostasis may be crucial for pancreatic disease progression.

## 2. Materials and Methods

This article is methodologically positioned as a comprehensive narrative review. The literature search was conducted using the PubMed and Google Scholar databases.

At the planning stage, we selected the most common pancreatic pathologies and trace elements with a documented or potential impact on pancreatic function. We focused on elements essential for normal pancreatic physiology (Fe, Cu, Co, I, Mn, Zn, Se), essential elements that become toxic in excess (Fe, Cu, Mn), potentially toxic elements (Co, Ag), and highly toxic heavy metals (Cd, Hg, Pb). The investigated diseases included DM, acute AP, CP, AI, and PC, predominantly represented by PDAC.

The search encompassed articles published between 1981 (the year of the first relevant publication on this topic) and 2026, with the vast majority of the included literature published within the last 10 years to reflect the most current state of knowledge. The search strategy utilized the following keywords and their combinations using the Boolean operator “AND”: “iron” OR “Fe”, “copper” OR “Cu”, “cobalt” OR “Co”, “iodine” OR “I”, “manganese” OR “Mn”, “zinc” OR “Zn”, “silver” OR “Ag”, “cadmium” OR “Cd”, “mercury” OR “Hg”, “lead” OR “Pb”, “selenium” OR “Se” AND “diabetes” OR “DM”, “acute pancreatitis” OR “AP”, “chronic pancreatitis” OR “CP”, “autoimmune pancreatitis” OR “AIP”, “pancreatic cancer” OR “PC”, “Pancreatic Ductal Adenocarcinoma” OR “PDAC”, and “treatment”. Only peer-reviewed articles written in English were considered for inclusion.

Following the initial database search, duplicate records were removed using reference management software and manual screening. The remaining articles underwent title and abstract screening based on predefined selection criteria. Studies were included if they directly evaluated the relationship between the specified trace elements (measured via dietary intake, blood/urine biomarkers, or tissue concentrations) and the physiological or pathological mechanisms of the aforementioned pancreatic diseases.

Although this is a narrative review rather than a formal systematic review, the included literature was rigorously evaluated for credibility and risk of bias. Rather than using standardized quantitative bias assessment tools, a qualitative evaluation was applied. Studies were assessed based on the robustness of their study design, adequate sample sizes, the presence of appropriate control groups, and whether confounding factors were properly adjusted for in clinical and epidemiological analyses. Emphasis was placed on original in vitro and in vivo experimental research, prospective cohort studies, and meta-analyses. We explicitly excluded non-peer-reviewed articles, case reports lacking broader mechanistic relevance, biased literature reviews, and studies whose findings had been unequivocally refuted by subsequent high-quality research.

In the subsections detailing the role of trace elements in specific pancreatic diseases, preference was given to original experimental and clinical studies to clearly distinguish between established clinical data and preclinical models. For the Section 1, comprehensive review articles and systematic reviews were preferentially utilized.

Following deduplication, screening, and critical quality assessment, a total of 330 studies met all criteria and were ultimately included in the final narrative synthesis.

## 3. The Influence of Trace Elements on the Physiological Function of the Pancreas

**Figure 2:**
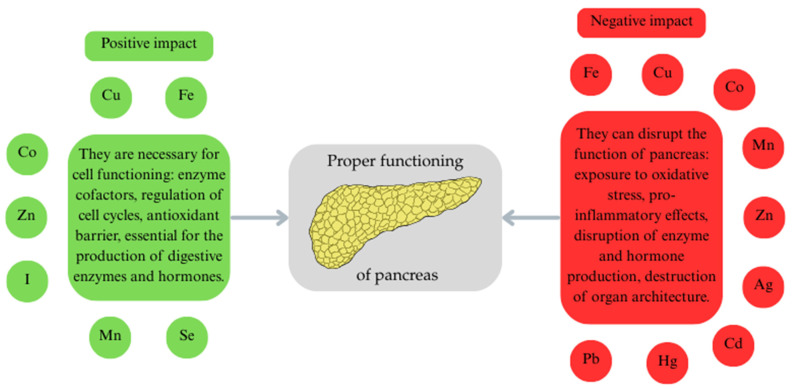
The influence of trace elements on pancreatic function. Abbreviations: Ag—Silver; Cd—Cadmium; Co—Cobalt; Cu—Copper; Fe—Iron; Hg—Mercury; I—Iodine; Mn—Manganese; Pb—Lead; Se—Selenium.

Trace elements are essential for the proper functioning of the pancreas, but in abnormal concentrations, they can damage it (Figure 2).

Fe is an essential element for pancreatic cells. In pancreatic β-cells, Fe is necessary for the proper production and secretion of insulin: it regulates insulin transcription. It is a component of iron–sulfur (Fe–S) clusters, proteins involved in insulin secretion after β-cell proliferation and differentiation. As a cofactor of prolyl and asparaginyl hydroxylase, Fe influences the response of β-cells to oxidative stress [20]. A 2020 study showed that β-cells, compared to α and δ-cells, exhibit higher expression of genes key to Fe import and storage, and that reduced Fe availability to β-cells resulted in reduced insulin secretion in mouse model. On the other hand, excessive exposure to too much of this element can lead to damage to β-cells [21]. In conditions associated with Fe overload, the exocrine function of the pancreas may also be impaired [22]. Cell damage as a result of excessive exposure to Fe may occur as a result of ferroptosis, a type of cell death caused by excessive Fe-dependent lipid peroxidation [23].

Cu, as a trace element, is also essential for the proper functioning of pancreatic cells. It is a component of Cu/ZnSOD, an enzyme involved in the removal of free radicals. Studies in a mouse model have shown that reduced dietary Cu intake, and thus reduced Cu body concentration, causes damage and reduces the number of pancreatic acinar cells, resulting in decreased digestive enzyme secretion [24,25]. However, at high concentrations, Cu can promote the formation of reactive oxygen species (ROS) via the Fenton reaction, in which Cu ions, like Fe ions, act as catalysts. It can increase oxidative stress in pancreatic cells [26,27].

Although Co, as a component of vitamin B12, is essential for proper body function, excessive exposure can be toxic to pancreatic cells. Studies in rats have shown that Co administration can inhibit insulin secretion by pancreatic islet cells by reducing the influx of Ca^2+^ into pancreatic islet β-cells [28,29].

Animal studies have demonstrated that in inflammatory conditions and oxidative stress, I has a protective effect on pancreatic cells, inhibits acinar fibrosis, insulin imbalance, and oxidative damage. It may perform this function by activating antioxidant pathways involving nuclear factor erythroid-2-related factor-2 (Nrf2) and peroxisome proliferator-activated receptor gamma (PPAR-γ). It is probably a direct activator of Nrf2, thereby inducing the expression of protective antioxidant enzymes, such as superoxide dismutase (SOD) type 1 and catalase (Cat). I also has the ability to bind to arachidonic acid and activate PPAR-γ, which has immunoregulatory, metabolic, and antioxidant effects. [30,31].

After soft tissues and the liver, the pancreas is the third-largest Mn-accumulating organ, containing approximately 5% of the body’s total Mn reserves [32]. Mn builds one of the main antioxidant enzymes, manganese superoxide dismutase (Mn-SOD), whose role is to protect against oxidative stress. The correct concentration of Mn and the proper function of Mn-SOD are important for pancreatic β-cells: the antioxidant function of this enzyme is essential for insulin secretion and survival [33]. Interestingly, studies on guinea pig pancreatic cells have shown that excessive exposure of pancreatic acinar cells to extracellular Mn can reduce pancreatic lipase secretion [34].

Another element essential for the proper functioning of the pancreas is Zn. It is necessary for the proper synthesis, structural stability, and storage of insulin in the granules of pancreatic β-cells. Zn is essential for the structural stability and storage of insulin hexamers within β-cell granules. This process takes place in Zn-rich secretory granules. Furthermore, Zn may also be involved in glucagon secretion by α-cells. Zn acts as a signalling ion in the intercellular space, causing the release of glucagon from pancreatic α-cells during glucose deficiency. Zn is also essential for the proper functioning of digestive enzymes produced by the pancreas: it participates in their metallisation, making their activity dependent on Zn. Zn deficiency disrupts this mechanism, which weakens the pancreas’s ability to secrete active digestive enzymes. Zn also builds one of the dismutases, thereby participating in the creation of defence against oxidative stress in pancreatic cells [35,36]. On the other hand, excess dietary Zn does not improve pancreatic function. A study conducted on chickens showed that an excessive supply of Zn led to changes in the structure of pancreatic acini and a decrease in the activities of amylase, lipase, trypsinogen, and chymotrypsinogen [37].

The effect of Ag on the pancreas is exerted through silver nanoparticles (AgNPs) and is essentially negative. In a mouse model, they caused structural disintegration of the pancreas, death of acinar cells and β-cells, and, as a consequence, decreased insulin secretion [38].

Cd is a metal that may accumulate in the pancreas of smokers. Its accumulation may cause damage to the acini of the exocrine pancreas. A study conducted on guinea pigs has shown that Cd, in the mechanism of maintaining intracellular Ca^2+^ concentration, mitochondrial depolarisation, and adenosine triphosphate (ATP) depletion, inhibits the secretion of pancreatic ductal fluid and bicarbonate (HCO_3_^−^) and reduces the expression of the apical cystic fibrosis transmembrane conductance regulator (CFTR) in ductal cells [39]. Cd also damages the endocrine part of the pancreas. This metal can accumulate in β-cells, disrupting their function and leading to their apoptosis. It acts through mechanisms including increased ROS and malondialdehyde (MDA) production, mitochondrial dysfunction, PARP cleavage, and activation of the caspase cascade: caspases 3, 7, and 9 [40,41]. The effect of Cd also affects α-cells—in their case, Cd disrupts glucagon synthesis and gene expression [42].

Similar to Cd, Hg levels are also elevated in smokers and affect the pancreas. In exocrine cells, Hg also inhibits the secretion of pancreatic duct fluid and bicarbonate, and reduces CFTR expression, via the same mechanism as Cd [39]. A study in a mouse model also demonstrated the effects of Hg on the endocrine part of the pancreas. Hg compounds disrupted β-cell function by exposing cells to oxidative stress, which was influenced by a reduction in the expression of mRNA genes, Nrf2, GPx, and NAD(P)H:quinone oxidoreductase 1 (NQO1), which are associated with the antioxidant barrier. Hg also promoted β-cell apoptosis through changes in the mRNA expression of anti-apoptotic genes: B-cell lymphoma 2 (Bcl-2), Myeloid Cell Leukaemia 1 (Mcl-1), and Mouse Double Minute 2 homolog (Mdm-2), and apoptotic genes: p53, caspase-3, and caspase-7. Furthermore, low doses of Hg inhibited phosphatidylinositol 3-kinase/protein kinase B (PI3K/Akt) pathway. These effects reduce insulin production by β-cells [43,44].

Reports on the effects of Pb on pancreatic function are scarce, but based on available data, it can be concluded that exposure to Pb impairs the endocrine part of the pancreas, causing insulin production disorders and inhibiting cell proliferation [45,46].

Se is an essential trace element that is a component of enzymes that form an antioxidant barrier: GPx, three thioredoxin reductases (TrxR), and methionine reductase-R-sulphoxide reductase B1 (MSRB1), as well as non-enzymatic selenoproteins, which also have antioxidant activity: selenoprotein P (SELENOP), selenoprotein W (SELENOW), selenoprotein K (SELENOK), and selenoprotein S (SELENOS). For this reason, Se deficiency has a toxic effect on pancreatic cells: in a study on male Yorkshire pigs, there was a decrease in serum insulin and glucagon concentrations and disruption of pancreatic islet cellular structure, induced by apoptosis [47]. Furthermore, Se supplementation increased insulin content and secretion in a study of isolated rat islets of Langerhans [48].

## 4. Pathophysiological Axes in Pancreatic Metallomics

The diverse pathological effects of trace elements in the pancreas converge on several common mechanistic axes that bridge the gap between DM, pancreatitis, and PC [49,50,51]. Rather than acting in isolation, these elements interact within integrated biological networks, dictating the progression from metabolic dysfunction and chronic inflammation to malignant transformation [51,52,53,54,55,56,57].

### 4.1. β-Cell Vulnerability and Endocrine Dysregulation

The endocrine function of the pancreas relies heavily on the precise homeostatic control of trace elements, and β-cells are uniquely vulnerable to metallomic imbalances due to their intrinsically low antioxidant capacity [58]. The structural stability, storage, and secretion of insulin within β-cell granules are fundamentally dependent on Zn, which facilitates the formation of Zn-insulin crystalline hexamers [35,36]. Intracellular Zn availability is tightly regulated by the zinc transporter 8 (ZnT8), and dysregulation of this transport severely compromises glucose metabolism [59,60,61].

Conversely, β-cells are highly susceptible to the accumulation of toxic heavy metals and the excess of essential elements [40,41,42,43,44]. Cd directly accumulates in pancreatic islets, where it interferes with calcium signaling, depletes cellular ATP, and activates Mitogen-activated protein kinase (MAPK)/JNK pathways, ultimately triggering caspase-dependent apoptosis and reducing β-cell mass and insulin production [40,62,63,64,65]. Pb exposure similarly impairs endocrine function, leading to decreased insulin levels, activation of glycogen synthase kinase-3β, and endoplasmic reticulum stress [66,67]. Furthermore, β-cells exhibit higher expression of genes involved in Fe import and storage than α- or δ-cells, reflecting their increased metabolic requirement for Fe [21,68]. However, this renders them exceptionally prone to Fe overload, which through oxidative stress inactivates critical transcription factors such as Pancreatic and duodenal homeobox 1 (PDX1) and V-maf musculoskeletal fibrosarcoma oncogene homolog A (MafA), thereby impairing insulin synthesis and secretion [69]. Additionally, Cu and Fe interact with human islet amyloid polypeptide (hIAPP), forming redox-active complexes that generate ROS, thereby disrupting cell-to-cell synchronization, reducing pulsatile insulin secretion, and promoting β-cell apoptosis [70,71,72,73,74].

### 4.2. Oxidative Stress and Mitochondrial Dysfunction

Oxidative stress and mitochondrial dysfunction represent a central pathophysiological axis across DM, pancreatitis, and PC [49,50,51]. At the core of this pro-oxidant environment are Fe and Cu, which are redox-active and act as potent catalysts in the Fenton reaction [26,27,58,75]. When present simultaneously in high concentrations, the labile pools of these trace elements drive non-enzymatic Fenton and Haber–Weiss chemistry, generating highly reactive hydroxyl radicals [50,75,76]. This specific Fenton-driven generation of excess ROS underlines the pathogenesis of diabetes and inflammation, amplifying nonspecific protein damage and disrupting cellular integrity [75]. In diabetes, excessive Fe entrance into the mitochondria depolarizes the organelle’s membrane potential, disrupting the electron transport chain and the energy supply required for insulin release [77]. In PDAC, Fenton-mediated oxidative stress synergizes with oncogenic Kirsten rat sarcoma viral oncogene homolog (KRAS) signaling, generating excessive mitochondrial ROS that overwhelms DNA repair pathways and promotes genomic instability, characterized by base modifications and DNA strand breaks [55,78,79,80]. However, PDAC cells successfully adapt to this stress by exploiting moderate Cu-induced ROS as a signaling mechanism to promote oncogenic pathways (e.g., MAPK and PI3K–Akt), driving tumor progression [51,54].

This oxidative burden is further amplified by exposure to Cd, Hg, and Pb [81,82,83]. Although not directly involved in Fenton-type redox chemistry, Cd provokes severe oxidative and nitrosative stress by depleting intrinsic antioxidant defenses, including GSH, SOD, and Cat [81,84,85,86]. MeHg exerts a similar toxic effect due to its high affinity for sulfhydryl and selenohydryl groups, triggering severe mitochondrial dysfunction and inducing functional Se deficiency [82].

Counteracting these pro-oxidant forces are Zn, Se, and Mn, which serve as critical components of the pancreatic antioxidant barrier [7,33,47]. Mn is a specific cofactor for mitochondrial Mn-SOD, and its reduced activity—frequently observed in PC tissues—accelerates oxidative DNA damage [55,87]. Zn functions as a key cofactor for Cu/Zn-SOD and stabilizes cellular membranes, whilst its deficiency, common in CP and DM, exacerbates lipid peroxidation [88,89,90,91,92]. Se is integral to GPx; however, excessive accumulation of Se in DM can lead to overactivation of GPx1, which scavenges hydrogen peroxide (H_2_O_2_) needed for physiological insulin signaling, paradoxically inducing insulin resistance [93,94].

### 4.3. Metal-Induced Cell Death Pathways: Ferroptosis and Cuproptosis

Trace elements are direct regulators of programmed cell death pathways, most notably ferroptosis and cuproptosis, which dictate the fate of pancreatic cells across inflammatory and malignant states [23,95,96]. Ferroptosis is a regulated, non-apoptotic form of cell death driven by Fe-dependent lipid peroxidation. In Fe-rich conditions, redox-active Fe catalyzes the non-enzymatic formation of lipid radicals strictly via Fenton chemistry. These lipid peroxides propagate chain reactions that disrupt membrane integrity [95]. In the context of AP and CP, Fe overload initiates ferroptosis, leading to the gradual loss of acinar cells, exacerbation of tissue necrosis, and the release of damage-associated molecular patterns (DAMPs) [50,76,97,98,99]. These DAMPs act as extracellular inflammatory mediators, creating a destructive feedback loop that sustains chronic inflammation and progressive fibrosis [98,100,101]. Cu further exacerbates this process in AP by directly binding to GPx4—the primary enzyme suppressing ferroptosis—and promoting its autophagic degradation [102,103].

In stark contrast, PDAC cells actively rewire their metabolism to acquire robust ferroptosis resistance. Despite accumulating vast amounts of intracellular Fe to support rapid proliferation and DNA synthesis, pancreatic cancer cells coordinately upregulate antioxidant defense systems, particularly GPx4 and the cystine/glutamate antiporter system Xc- (SLC7A11). This adaptation decouples Fe accumulation from ferroptotic vulnerability, conferring resistance to chemotherapy and radiotherapy [104,105,106].

Cuproptosis, a recently identified Cu-dependent form of cell death, involves intracellular Cu accumulation, mitochondrial protein lipoylation, loss of Fe-S clusters, and impairment of the ubiquitin–proteasome system [96,107]. While excess Cu can induce cuproptosis and acinar cell damage in CP [108], PDAC cells exhibit a unique sensitivity to Cu-induced mitochondrial dysfunction [32,109]. Nevertheless, cancer cells often initiate an autophagy-mediated survival mechanism to limit the efficacy of Cu-induced cytotoxicity, highlighting the dual role of these metals as both essential metabolic cofactors and potent cytotoxic agents [110,111].

### 4.4. The Ductal and Inflammatory Axis

The exocrine pancreas is highly susceptible to structural remodeling and functional impairment driven by a specific secretory–inflammatory axis [39,112]. Heavy metals, particularly Cd and Hg—which are significantly elevated in smokers—accumulate in pancreatic tissue and acutely disrupt ductal physiology [39,113]. Both Cd and Hg severely inhibit pancreatic ductal fluid and bicarbonate (HCO_3_^−^) secretion and reduce the apical expression and localization of the CFTR in ductal cells. This loss of ductal secretory capacity results in a highly acidic, viscous microenvironment, a key feature of CP pathology that contributes to premature proenzyme activation and acinar atrophy [39].

**Figure 3:**
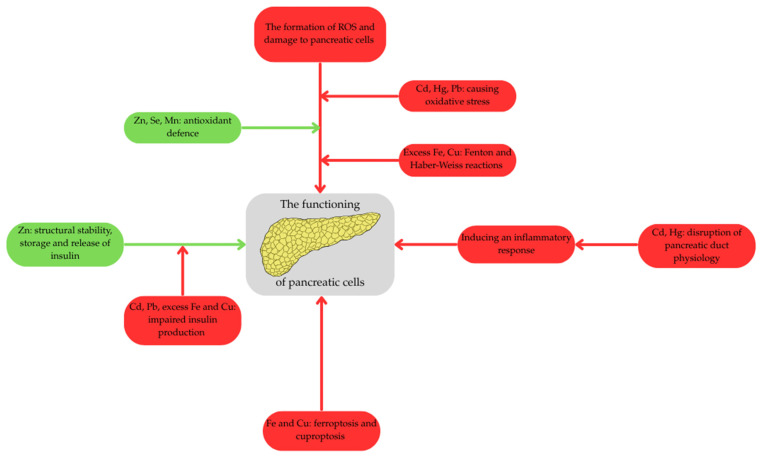
Integration of pathophysiological axes in pancreatic metallomics. Abbreviations: Cd—Cadmium; Co—Cobalt; Cu—Copper; Fe- Iron; Hg—Mercury; Mn- Manganese; Pb—Lead; Se—Selenium; ROS—Reactive Oxygen Species; Zn—Zinc.

Following ductal impairment, trace element dyshomeostasis aggressively drives chronic inflammation and tissue remodelling [112,114]. Cd activates canonical inflammatory pathways, notably the Nuclear factor kappa B (NF-κB) pathway, leading to the massive upregulation of pro-inflammatory cytokines such as tumor necrosis factor alpha (TNF-α), Interleukin-6 (IL-6), and IL-1β [112,114,115,116,117,118]. Simultaneously, Co accumulation stabilizes hypoxia-inducible factor-1α (HIF-1α) even under normoxic conditions, further exacerbating the inflammatory response and fibrotic remodeling [119]. This relentless inflammatory loop, characterized by excessive extracellular matrix deposition and stellate cell activation (driven by Fe-induced oxidative stress), not only leads to the irreversible structural collapse seen in CP but also generates the dense desmoplastic stroma characteristic of PDAC [56,120]. This stroma subsequently facilitates immune evasion, supports tumor invasion, and creates a formidable physical barrier against effective chemotherapeutic drug penetration [56,120,121]. In Figure 3 we summarized the common pathophysiological axes in pancreatic metallomics.

## 5. The Influence of Trace Elements on Pancreatic Pathology

Trace elements affect not only physiology but also pancreatic diseases. In this chapter, we present the effects of trace elements: Fe, Cu, Co, I, Mn, Zn, Ag, Cd, Hg, Pb, and Se on pancreatic diseases: DM, pancreatitis, and PC. We define the role of these elements in the pathogenesis and development of diseases, as well as their potential therapeutic applications. In the manuscript, we cite studies conducted in animal and cell models; appropriate caution should be exercised when interpreting and transferring data to a human model, underscoring the need for further research. It is important to note that the depth of the mechanistic discussion in the following sections inherently varies among the trace elements, directly reflecting the current state of the scientific literature. Essential, redox-active elements such as Fe and Cu have been extensively studied, yielding deep, well-established mechanistic insights into their roles in ferroptosis, cuproptosis, and oxidative stress. Conversely, the available literature regarding toxic or exploratory elements like Co, Ag, Pb, and I is considerably sparser. Consequently, discussions concerning these elements are relatively brief and focus primarily on phenomenological observations from preclinical models, reflecting the current limitations of pancreatic metallomics research.

### 5.1. The Effect of Trace Elements on Diabetes

The first of the described diseases of the pancreas is DM, which is a metabolic disorder characterised by the inappropriate elevation of blood glucose levels. This condition encompasses several categories, including type 1 diabetes mellitus (T1DM), type 2 diabetes mellitus (T2DM), maturity-onset diabetes of the young (MODY), gestational diabetes mellitus (GDM), neonatal diabetes, and secondary forms resulting from endocrinopathies and corticosteroid therapy, among other causes. The primary subtypes of DM are T1DM and T2DM, which typically arise from defective insulin secretion and/or impaired insulin action, respectively. T1DM primarily manifests in pediatric and adolescent populations, whereas T2DM is generally observed in middle-aged and older individuals, often associated with lifestyle and dietary factors contributing to sustained hyperglycemia. The pathophysiological mechanisms underlying T1DM and T2DM are markedly distinct, leading to differences in etiology, clinical presentation, and therapeutic approaches [122,123].

T1DM results from the autoimmune destruction of pancreatic β cells, leading to an absolute insulin deficiency. Conversely, T2DM is characterized by chronic hyperglycemia, resulting from a combination of insulin overproduction, peripheral insulin resistance, and impaired glucose utilization. Persistent hyperglycemia induces nonenzymatic glycation of proteins and lipids, a process quantitatively assessed by the glycosylated hemoglobin (HbA1c) test. Glycation end products contribute to microvascular damage in critical tissues, such as the retina, kidneys, and peripheral nerves. Elevated glucose concentrations accelerate this process, which underlies the pathogenesis of diabetic micro and macrovascular complications, including retinopathy, nephropathy, neuropathy, atherosclerosis, and ischemic heart disease. These complications are associated with preventable adverse outcomes such as blindness, renal failure requiring dialysis, and limb amputation [124,125].

Recent studies link the pathogenesis of DM to numerous trace elements.

#### 5.1.1. The Effect of Fe on Diabetes

Fe has been shown to influence DM types 1 and 2 and GDM.

According to a meta-analysis by Liu et al. [126], multiple epidemiological studies have demonstrated a positive association between elevated serum ferritin levels and an increased risk of T2DM. Although ferritin concentrations are influenced by several confounding factors—including sex, ethnicity, age, dietary Fe intake, and systemic inflammation—accumulating evidence suggests that increased body Fe burden contributes to the pathogenesis of DM [126]. This concept is further supported by the clinical manifestations of Fe overload disorders, such as hereditary hemochromatosis or thalassemias, in which DM is a well-recognized complication. Notably, in hereditary hemochromatosis, pancreatic β-cells appear to be preferentially damaged compared with α- or δ-cells [127].

Fe uptake into pancreatic β-cells occurs through two main mechanisms. The first is receptor-mediated transport involving transferrin-bound iron (TBI), which binds to transferrin receptor 1 (TfR1) on the cell surface. Following internalization of the TfR1–transferrin complex, Fe is released within endosomes and transported into the cytosol. The second mechanism, described more recently, involves the uptake of non-transferrin-bound iron (NTBI). In circulation, NTBI exists in various low-molecular-weight forms. NTBI uptake is mediated predominantly by the Zn transporter Zrt- and Irt-like protein 14 (ZIP14) (SLC39A14), which is expressed on the membranes of pancreatic islet cells and contributes to Fe loading under conditions of Fe excess [128,129,130].

Once internalized, intracellular Fe is largely sequestered by ferritin, thereby limiting its redox activity and potential cytotoxic effects.

Fe efflux from cells is primarily regulated by ferroportin (Ferroportin 1 (FPN1); SLC40A1), the only known cellular Fe exporter. Pancreatic islets exhibit low ferroportin immunoreactivity, suggesting limited Fe export capacity. However, islet cells express hephaestin, a multicopper ferroxidase that stabilizes ferroportin at the plasma membrane and catalyses the oxidation of ferrous Fe (Fe^2+^) to ferric Fe (Fe^3+^), a prerequisite for Fe loading onto transferrin in the circulation [68,131].

As detailed in Section 4, Fe overload impairs β-cell function and viability through multiple mechanisms linked to excessive ROS production and ferroptosis [58,69,77,95,120,132]. In insulin-resistant states, hIAPP is overproduced and co-secreted with insulin, creating conditions that favour its misfolding and aggregation within pancreatic islets. Elevated Fe availability increases levels of redox-active heme, which has been shown to bind specifically to hIAPP via His18, forming a stable heme–hIAPP complex. Unlike canonical heme-containing enzymes, this complex lacks protective peroxidase or Cat activity and instead facilitates one-electron reduction of molecular oxygen, generating ROS such as hydrogen peroxide. Given the limited antioxidant defences of pancreatic β-cells, this oxidative burden promotes activation of apoptotic pathways. Moreover, oxidative stress further accelerates hIAPP misfolding and oligomerisation, establishing a self-amplifying cycle of β-cell damage that contributes to progressive β-cell loss and the development of overt T2DM [70,71].

Chronic hyperglycemia has been shown to upregulate ZIP14 (SLC39A14) expression, suggesting a mechanistic link between elevated NTBI levels and DM. However, small interfering ribonucleic acid (siRNA)-mediated knockdown of ZIP14 results in only approximately a 50% reduction in NTBI uptake, indicating the involvement of additional, as yet incompletely characterized transporters. Notably, elevated NTBI concentrations have been detected in several pathological states, often in the absence of marked transferrin saturation. In individuals with DM, NTBI can be detected at transferrin saturation levels below 60%, underscoring its potential pathogenic relevance even in the absence of classical Fe overload [20].

Several studies noted a correlation between inappropriate hepcidin production and DM. Interestingly, both low and high hepcidin levels were noted before in patients with DM. Disorders such as thalassemia or hereditary hemochromatosis are mostly connected with low levels of hepcidin, while metabolic syndrome usually coexists with elevated levels of hepcidin. Studies also show that Fe overload is associated with decreased adiponectin, an insulin-sensitising adipokine [133].

The hepcidin–ferroportin axis is the central regulator of systemic Fe homeostasis, as demonstrated by extensive mouse model data establishing hepcidin as the master controller of dietary Fe absorption and macrophage Fe release. Genetic disruption of key hepcidin regulators—including hemochromatosis gene (HFE), hemojuvelin (HJV), transferrin receptor 2 (TfR2), and TMPRSS6—recapitulates human Fe overload or Fe-deficiency phenotypes in vivo, with hepatic hepcidin expression predominantly governed by endothelial Bone morphogenetic protein (BMP)2/BMP6-driven BMP–mothers against decapentaplegic (SMAD) signalling. While hepatocytes mediate systemic regulation, Fe handling in macrophages, enterocytes, and other tissues fine-tunes Fe distribution.

Mouse models of severe Fe overload (e.g., Hamp^−/−^, Hjv^−/−^, and ferroportin p.C326S) reveal a key translational limitation: despite massive Fe accumulation in organs such as the liver and pancreas, classical human complications, such as DM, cirrhosis, or cardiomyopathy, do not consistently develop. This dissociation between Fe burden and organ pathology is attributed to the relative resistance of murine tissues to oxidative stress-induced injury, underscoring that Fe overload alone is insufficient to drive disease. In DM, Fe toxicity likely arises from the convergence of altered hepcidin signalling with metabolic stressors—such as inflammation, hyperglycemia, and limited antioxidant capacity—rather than from extreme Fe accumulation per se. Mouse models with genetic disruptions in the hepcidin–ferroportin axis provide insights into Fe overload, though they do not always mirror human diabetic phenotypes; further research is needed to prove this hypothesis [134].

A growing body of evidence indicates that maternal Fe status, particularly elevated serum ferritin, is associated with increased GDM risk, and that higher dietary heme Fe intake may further elevate risk independent of total Fe intake. Observational studies suggest high-dose Fe supplementation may be associated with a greater incidence of GDM in Fe-replete women. However, randomized trials report inconsistent findings, potentially due to differences in dosing, duration, and baseline Fe status [134,135]. Mechanistically, excess Fe may exacerbate oxidative stress and insulin resistance in pregnancy, contributing to impaired β-cell compensation characteristic of GDM [135,136].

In summary, serum ferritin levels are correlated with an increased risk of T2DM. It should be noted that high ferritin levels may also be influenced by systemic inflammation [126]. High ferritin levels and high heme Fe intake in pregnant women increase the risk of GDM by exacerbating insulin resistance and oxidative stress [135,136].

#### 5.1.2. The Effect of Cu on Diabetes

Meta-analyses showed increased serum Cu levels in patients with DM, regardless of DM type, compared with healthy controls [137]. It may be due to chronic inflammation in patients with DM, resulting from increased levels of acute-phase proteins, including ceruloplasmin [138].

Multiple clinical studies have shown a positive association between serum Cu levels and markers of glycemic control, most notably HbA1c, with the strongest correlations observed in individuals with poorly controlled DM [139,140]. Importantly, when confounding factors such as genetic variability are minimised in longitudinal analyses, changes in HbA1c positively correlate with changes in ceruloplasmin concentrations, supporting the concept that chronic hyperglycemia itself promotes increased ceruloplasmin and Cu levels [141,142]. In line with this observation, patients with well-controlled glycemia (HbA1c < 6.5%) exhibit Cu concentrations comparable to those of healthy controls, whereas those with poor glycemic control exhibit significantly elevated Cu levels [143]. A reduction in serum HbA1c may lead to decreased Cu levels, thereby improving glycemic control and reducing the risk of complications [139,140].

Hyperglycemia promotes non-enzymatic glycation of proteins, generating glycated structures with enhanced affinity for transition metals, including Cu. Importantly, protein-bound Cu retains redox activity and can catalyse the formation of ROS, thereby intensifying oxidative stress in diabetic tissues. This mechanism provides a molecular link between hyperglycemia, Cu accumulation, and oxidative damage [141]. As previously mentioned in Section 4, Cu-dependent oxidative stress and cuproptosis are central mechanisms linking hyperglycemia to cellular injury in DM [72,73,74,75,96,107,140].

Elevated Cu and ceruloplasmin levels have been implicated in the development of diabetic vascular complications. Chronic hyperglycemia-induced increases in ceruloplasmin and Cu are associated with heightened cardiovascular risk, likely mediated through oxidative damage to endothelial cells and promotion of lipid peroxidation [141,142]. Clinical and experimental data indicate that lowering HbA1c is associated with reduced serum Cu levels, suggesting that improved glycemic control may mitigate Cu-driven oxidative injury [139,140].

Cu dysregulation has also been implicated in GDM. Meta-analyses and large observational studies demonstrate higher circulating Cu concentrations in women with GDM, particularly during the third trimester and among Asian populations [144,145]. Cu levels rise with gestational age, largely due to estrogen-induced increases in ceruloplasmin synthesis, which may exacerbate Cu-related oxidative stress during pregnancy [146]. Dose–response analyses further support a positive association between circulating Cu and GDM risk [147].

Epidemiological studies indicate that high dietary Cu intake and elevated serum Cu concentrations are associated with an increased risk of T2DM, particularly in men and in individuals with comorbid hypertension [148,149]. While excessive Cu appears deleterious, Cu deficiency also disrupts antioxidant defences and metabolic homeostasis. Current evidence suggests that Cu intake should remain within recommended dietary allowance levels to avoid both deficiency and toxicity [26].

#### 5.1.3. The Effect of Co on Diabetes

Although experimental animal models suggest that low to moderate doses of cobalt chloride (CoCl_2_) may exert certain protective effects—such as reducing gluconeogenesis, decreasing lipid peroxidation, and attenuating diabetic nephropathy through HIF-1α stabilization [150,151,152], the clinical relevance of Co in DM pathogenesis remains strictly exploratory. Human studies assessing Co status in DM are highly heterogeneous and inconclusive [152,153,154]. While some reports indicate lower Co concentrations in the serum and saliva of diabetic patients, others show positive or non-robust associations [155]. Furthermore, excessive Co exposure is known to promote mitochondrial dysfunction and ROS generation [156]. Given the lack of consistent clinical validation and the reliance on preclinical models, further robust clinical studies are required to determine whether altered Co status is a causal factor or merely a secondary consequence of diabetic pathology [156,157].

#### 5.1.4. The Effect of I on Diabetes

The role of I in the pathogenesis of DM is complex and primarily indirect, mediated almost entirely through its essential function in thyroid hormone synthesis [158]. Thyroid dysfunction, which is highly prevalent among DM patients, significantly impacts metabolic control: hyperthyroidism can reduce peripheral insulin sensitivity, while hypothyroidism impairs glucose disposal [158,159,160]. In clinical cohorts, urinary I concentration has been inversely correlated with fasting glucose and insulin resistance indices [161]. In the context of GDM, a lower placental I load has been associated with borderline significant increases in GDM risk [162].

However, population studies reveal conflicting associations between urinary I and DM risk, heavily influenced by baseline dietary sufficiency, genetic susceptibility, and the presence of non-thyroidal illness syndrome in advanced DM [163,164,165,166,167]. While some experimental models suggest postpartum I supplementation might exert immunomodulatory effects in T1DM [168], direct biochemical effects of I on pancreatic function remain speculative. Therefore, current evidence does not support I as a direct driver of pancreatic metallomic dysregulation.

#### 5.1.5. The Effect of Mn on Diabetes

As detailed in Section 4.2, Mn plays a fundamental role in regulating pancreatic oxidative stress as a critical cofactor for mitochondrial Mn-SOD. Experimental studies confirm that physiological Mn levels enhance insulin secretion and improve glucose homeostasis, whereas excessive exposure disrupts mitochondrial function and carbohydrate metabolism [49,169,170,171,172,173].

At the epidemiological level, evidence linking Mn status to T2DM remains inconsistent [170,174]. Current clinical data suggest a U-shaped relationship, where both excessively low and high Mn concentrations increase DM risk [171,175]. While lower Mn concentrations in blood or hair have been observed in some diabetic populations [176,177,178], the clinical interpretation is complicated by consistently reported sex-specific differences [179,180]. Higher dietary Mn intake is associated with a significantly reduced risk of T2DM in women but not in men, a discrepancy potentially driven by differences in Mn absorption, hormonal influences, and competitive interactions between Mn and Fe [171,179,180].

In specific diabetes subtypes, elevated serum Mn levels have been associated with increased risk of GDM [172]. Conversely, evidence regarding T1DM remains highly limited, with most studies reporting a positive correlation between Mn and total antioxidant status in pediatric patients [181].

Overall, while the biological necessity of Mn in pancreatic function is clear, its clinical relationship with DM is complex, U-shaped, and sex-dependent, warranting further longitudinal investigation rather than immediate therapeutic application.

#### 5.1.6. The Effect of Zn on Diabetes

Unlike trace elements for which the evidence is predominantly preclinical data, the role of Zn in DM is supported by robust clinical, epidemiological, and genetic evidence. As detailed in Section 4.1 and Section 4.2, Zn is indispensable for insulin synthesis and storage within secretory granules via the ZnT8 transporter, and it acts as a critical cofactor for antioxidant defense (Cu/Zn-SOD) [59,60,61,88,182].

Clinically, disturbances in Zn homeostasis are evident across DM subtypes. Patients with T1DM exhibit significantly reduced serum Zn concentrations that correlate negatively with HbA1c levels, suggesting an association between Zn deficiency and poor glycemic control [183]. In T2DM, the relationship is more complex; chronic hyperglycemia drives hyperzincuria (increased urinary Zn excretion), further depleting systemic Zn stores and exacerbating oxidative stress [184,185,186]. While some meta-analyses demonstrate an inverse relationship between moderate dietary Zn intake and T2DM risk [184,187], other systematic reviews report no significant association [188], and excessively high Zn levels have even been positively associated with disease risk [187].

Regarding GDM, higher Zn exposure is generally associated with a reduced risk [189]. Meta-analyses confirm that women with GDM, particularly in Asian cohorts during the second trimester, show significantly lower serum Zn concentrations compared to healthy pregnant women [190].

Therapeutically, intervention studies demonstrate clear clinical benefits. Meta-analyses confirm that Zn supplementation in T2DM patients improves glycemic control, lowers fasting plasma glucose, and decreases systemic inflammation (lowered CRP) [191]. Although some studies report no protective association [187], the clinical consensus strongly positions Zn deficiency as a major, actionable factor in DM progression [184,185,186,191].

#### 5.1.7. The Effect of Cd on Diabetes

Cd is a well-established environmental diabetogen [192,193]. As detailed into Section 4.1 and Section 4.2, Cd directly induces β -cell apoptosis by disrupting calcium signaling and severely depleting intrinsic antioxidant defenses [40,62,63,64,65,81,84,85,86].

Epidemiologically, urinary and blood Cd concentrations are strongly correlated with elevated fasting glucose, prediabetes, and overt DM [192,193]. Beyond direct pancreatic toxicity, Cd drives systemic metabolic dysfunction by upregulating pro-inflammatory cytokines (TNF-α, IL-6) and disrupting peripheral insulin sensitivity [117,118]. In adipose tissue, Cd decreases Glucose transporter type 4 (GLUT-4) expression and downregulates essential adipogenic transcription factors (PPARγC/EBPα), thereby diminishing the secretion of insulin-sensitizing adipokines like adiponectin [117,194,195,196]. In the liver, Cd exposure augments gluconeogenic enzymes, collectively compounding hyperglycemia and linking environmental metal exposure to the obesity–diabetes phenotype [197,198].

#### 5.1.8. The Effect of Hg on Diabetes

Epidemiological evidence linking Hg to T2DM remains controversial, with studies showing heterogeneous correlations between systemic Hg levels and disease prevalence [199,200,201,202,203,204,205]. However, analysis of red blood cell (RBC)-bound Hg, which has a significantly longer half-life, has shown a positive association with T2DM and highlights specific dietary patterns, such as the consumption of fried fish and shellfish, as major exposure routes [206,207,208,209].

The clinical evidence is considerably stronger for GDM, where elevated whole-blood and RBC-Hg levels are unanimously associated with increased risk, particularly in the second trimester [189,210,211,212]. Mechanistically, as described in Section 4.2, methylmercury (MeHg) triggers profound mitochondrial dysfunction and functional Se deficiency by degrading GPx1 [82]. This results in decreased GLUT-4 activity and pronounced insulin resistance [61,213,214]. Interestingly, some meta-analyses suggest a paradoxical protective effect of low-dose Hg in men via transient Nrf2 activation, though this compensatory mechanism requires further clinical validation [82,205].

#### 5.1.9. The Effect of Pb on Diabetes

While preclinical models demonstrate that Pb effectively disrupts insulin secretion through endoplasmic reticulum stress (Section 4.1) and elevates ROS levels (Section 4.2) [66,83,215], epidemiological data in humans remain mixed [216,217,218,219]. The discrepancy largely depends on the biomarker used; urinary Pb levels, which better reflect chronic exposure, show more consistent positive associations with impaired fasting glucose and T2DM than blood Pb levels [108,216,217,218,219]. Clinically, co-exposure to Pb and Cd is particularly detrimental, as it is linked to hyperglycemia, reduced kidney function, and albuminuria, significantly worsening diabetic nephropathy in T2DM [83,108]. This variability across studies—arising from differences in exposure assessment, co-exposures, and individual susceptibility—underscores the critical need for longitudinal studies focusing on chronic heavy metal mixtures rather than isolated acute exposures to validate these relationships in human populations.

#### 5.1.10. The Effect of Se on Diabetes

Se possesses a uniquely narrow optimal therapeutic window, with both deficiency and excess drastically impacting glucose homeostasis [220,221,222]. As detailed in Section 4.2, the adverse effects of high Se intake are driven by the overactivation of GPx1, which excessively scavenges H_2_O_2_ required for physiological insulin receptor signalling, thereby precipitating insulin resistance [93,94,223,224].

Clinically, this manifests as a non-linear, U-shaped relationship, where individuals in the highest serum Se categories exhibit a significantly elevated risk of T2DM [220,221,222,225]. Notably, while observational data consistently link high Se intake to increased DM risk, randomized controlled trials (RCTs) of Se supplementation have not consistently confirmed an increased DM incidence, suggesting complex underlying metabolic interactions [226].

In stark contrast, GDM is robustly associated with Se deficiency. Meta-analyses confirm that women with GDM display significantly lower Se levels, particularly in the third trimester and in populations with grain-based diets (e.g., Asian and African cohorts) [143,227,228,229,230,231]. For this specific demographic, interventional evidence suggests that targeted Se supplementation can significantly reduce fasting plasma glucose and improve neonatal outcomes [232]. This highlights the critical need to distinguish between T2DM (where excess is a risk) and GDM (where deficiency is a risk) when considering Se in clinical practice.

A summary of the role of trace elements in DM is given in Table 1.

**Table 1:** The effect of trace elements on diabetes. The use of “→” denotes causality in the mechanism of the element’s influence on the disease, a down arrow indicates a decrease and an up arrow indicates an increase.

Trace Element	Effect on the Disease	Mechanism of Influence	Additional Information
Iron (Fe) [20,58,68,69,70,71,77,95,120,126,127,128,129,130,131,132,133,134,135,136]	Negative	Fe overload → excessive ROS via Fenton reaction → oxidative stress → mitochondrial dysfunction and β-cell damage → impaired insulin secretion	Linked to hemochromatosis; promotes ferroptosis and hIAPP aggregation; β-cells are especially sensitive due to low antioxidant capacity
Copper (Cu) [26,72,73,74,75,96,107,137,139,140,141,142,143,144,145,146,147,148,149]	Mainly negative	Hyperglycemia → protein glycation → Cu binding → ROS generation → oxidative damage; Cu also interacts with hIAPP → mitochondrial dysfunction and β-cell apoptosis	Strong correlation with HbA1c; involved in “cuproptosis”; contributes to vascular complications
Cobalt (Co) [150,151,152,153,154,155,156,157]	Mixed	Low/moderate levels → ↓ gluconeogenesis, ↓ lipid peroxidation, activation of HIF-1α; high levels → ROS production and mitochondrial dysfunction	Animal studies show protective effects; human data are inconsistent
Iodine (I) [158,159,160,161,162,163,164,165,166,167,168]	Mixed	I imbalance → altered thyroid hormones → changes in insulin sensitivity and glucose metabolism	Both deficiency and excess may increase risk, linked to thyroid disorders common in diabetes
Manganese (Mn) [49,169,170,171,172,173,174,175,176,177,178,179,180,181]	Mixed (U-shaped)	Cofactor of Mn-SOD → reduces ROS; deficiency → impaired insulin secretion; excess → mitochondrial dysfunction and oxidative stress	Sex-specific differences; interaction with Fe metabolism
Zinc (Zn) [59,60,61,88,182,183,184,185,186,187,188,189,190,191]	Mainly positive	Stabilizes insulin in secretory granules; regulates insulin secretion; antioxidant (via Cu/Zn-SOD, metallothionein); improves insulin sensitivity	Zn deficiency linked with poor glycemic control; hyperzincuria common; supplementation may improve HbA1c
Cadmium (Cd) [40,62,63,64,65,81,84,85,86,117,118,192,193,194,195,196,197,198]	Negative	Accumulates in β-cells → disrupts Ca^2+^ signaling → mitochondrial dysfunction → apoptosis; indirectly increases ROS and inflammation → insulin resistance	Increases pro-inflammatory cytokines (TNF-α, IL-6); impairs GLUT4 and adipocyte function
Mercury (Hg) [61,82,189,199,200,201,202,203,204,205,206,207,208,209,210,211,212,213,214]	Mixed	MeHg → binds sulfhydryl groups → depletes glutathione → oxidative stress → β-cell damage and apoptosis; ↓ GLUT4 → insulin resistance	Stronger evidence for GDM risk; fish consumption is main exposure source; possible weak protective effect in men (unclear mechanism)
Lead (Pb) [66,83,108,215,216,217,218,219]	Mainly negative	Increases ROS and inflammation; disrupts insulin signaling and secretion; ↑ gluconeogenesis → hyperglycemia	Epidemiological results inconsistent; chronic exposure more relevant (urinary Pb)
Selenium (Se) [93,94,143,220,221,222,223,224,225,226,227,228,229,230,231,232]	Mixed (U-shaped)	Excess Se → overactive selenoproteins (GPx1) → excessive ROS removal → impaired insulin signaling; deficiency → weak antioxidant defense	High levels linked to T2DM; deficiency common in GDM; narrow optimal range

Abbreviations: Cd—Cadmium; Co—Cobalt; Cu—Copper; Fe—Iron; Hg—Mercury; hIAPP—Human islet amyloid polypeptide; I—Iodine; Mn—Manganese; Mn-SOD—Manganese superoxide dismutase; Pb—Lead; ROS—Reactive oxygen species; Se—Selenium; TNF-α—Tumor necrosis factor alpha; Zn—Zinc; IL-6—Interleukin 6; GLUT4—Glucose transporter type 4; GPx1—Glutatione peroxidise 1; T2DM—Type 2 diabetes mellitus; GDM—Gestational diabetes mellitus; MeHg—Methylmercury; HIF-1α—Hypoxia-inducible factor-1 alpha.

### 5.2. The Effect of Trace Elements on Pancreatitis

The second of the pancreatic conditions discussed, which is influenced by trace elements, is pancreatitis (see Table 2).

AP is a disease involving sudden inflammation of the pancreas. The release of active pancreatic enzymes into the bloodstream and the stimulation of inflammatory cytokine production are key events in the development of the inflammatory cascade, which leads to systemic inflammatory response syndrome. It is one of the most common gastroenterological conditions requiring hospital treatment, with a mortality rate of 30–40% in severe cases involving organ failure or pancreatic necrosis. Most patients experience the oedematous form, which is mild, self-limiting, and uncomplicated. In 10–20% of cases, a severe necrotic form develops with a fatal prognosis. The most common causes are gallstones and alcohol. AP may also occur as a complication after endoscopic retrograde cholangiopancreatography (ERCP) and as a complication after elective pancreatic surgery. Several recent publications confirm the occurrence of AP after pancreatic resections as an event triggering other postoperative complications, such as pancreatic fistula and bleeding. One of the most serious complications is acute kidney injury [233,234].

CP is a progressive, irreversible inflammatory disease of the pancreas, leading to gradual fibrosis of the parenchyma and loss of exocrine and endocrine. Clinically, CP manifests primarily as chronic abdominal pain, pancreatic insufficiency with accompanying digestive disorders, and the development of secondary type 3c DM. The aetiology of the disease is complex, with the most common risk factors in adults being alcohol abuse and smoking, although genetic and autoimmune factors and anatomical abnormalities of the pancreatic ducts also play a significant role. Diagnosis is based on clinical presentation and imaging studies, such as computed tomography, magnetic resonance imaging, with magnetic resonance cholangiopancreatography (MRCP), and endoscopic ultrasonography, which allow assessment of structural and ductal changes. Treatment of CP is symptomatic and includes elimination of risk factors, pain control, enzyme replacement therapy, and treatment of metabolic complications, and in selected cases, endoscopic or surgical interventions [235,236].

AIP is a rare disease. Diagnosis is difficult and should be based on comprehensive clinical, radiological, serological, and pathological evaluation. Two types have been identified: type 1 AIP, associated with immunoglobulin G4 (IgG4)-related disease, and type 2 AIP, a pancreas-specific disease unrelated to IgG4. Although their pathophysiology differs, both types respond well to steroid therapy. The standard treatment is oral corticosteroids. Rituximab is used for remission induction and maintenance in relapsing AIP-1. In selected patients, immunomodulators such as azathioprine are used to maintain remission [237].

#### 5.2.1. The Effect of Fe on Pancreatitis

As comprehensively detailed in Section 4, Fe overload and the subsequent induction of ferroptosis are central to the pathogenesis of AP and CP [50,76,100,101].

In experimental models of AP, labile Fe accumulation promotes lipid peroxidation (partially via lipoxygenase and cytochrome P450 activity) and the release of DAMPs from ferroptotic acinar cells [98,99,238,239]. These DAMPs establish a destructive extracellular feedback loop that exacerbates the inflammatory response [98]. Furthermore, Fe overload in these models has been shown to induce pyroptosis via Tom20 oxidation and gasdermin E cleavage [97]. Studies using cerulein-induced pancreatitis in mice clearly demonstrate that Fe administration directly exacerbates acinar cell death and increases the production of pro-inflammatory cytokines such as IL-1β and IL-6 [99,240].

In the context of CP, preclinical models indicate that secondary Fe overload leads to prominent Fe deposition in acinar cells, severely disrupting cellular homeostasis. Chronic Fe exposure overwhelms protective enzymes (such as GPx4, SLC7A11, and SOD) and sustains chronic inflammation, marked by increased infiltration of lymphocytes (anti-CD3), neutrophils (anti-CD11b), and macrophages (anti-F4/80), alongside a notable reduction in the anti-inflammatory cytokine IL-10. Ultimately, this excessive inflammatory activation and ongoing ferroptotic cell death drive extensive collagen deposition, leading to progressive acinar atrophy and the irreversible structural fibrosis characteristic of advanced CP [100,101].

While the mechanistic links between Fe-induced ferroptosis and pancreatic tissue destruction are biologically plausible, it is important to note that these findings are currently predominantly derived from murine models of AP and CP [99,100,101,240]. Further translational studies are required to fully validate these specific immune and fibrotic responses in human pancreatitis.

#### 5.2.2. The Effect of Cu on Pancreatitis

As delineated in Section 4.2 and Section 4.3, Cu exerts a paradoxical dual role in the pancreas, acting both as a crucial antioxidant cofactor and a potent driver of programmed cell death [241,242,243].

Physiologically, Cu is essential for neutralizing ROS as a cofactor for Cu/Zn-SOD1, the Cu chaperone for SOD (CCS), and Antioxidant 1 [92,241,244]. Pathologically, as discussed in Section 4.3, excess Cu promotes ferroptosis in AP by directly binding to GPx4 (at cysteines C107 and C148), leading to its autophagic degradation [102,103]. In vitro studies in human cell lines confirm that Cu chelation attenuates erastin-induced cytotoxicity, highlighting its specific role in promoting ferroptosis [102]. Furthermore, excess Cu induces cuproptosis, destabilizing Fe-dependent proteins and driving acinar cell death, a process particularly relevant to the fibrotic progression of CP [245,246].

Clinically, patients hospitalized for AP exhibit significantly elevated blood Cu levels, along with increased markers of inflammation (IL-6, hs-CRP) and lipid peroxidation (MDA) [102]. Additionally, disturbances in the serum Cu/Zn ratio can shift the balance towards pro-oxidative processes, sustaining chronic inflammation [92]. Therefore, modulating Cu levels (e.g., via chelation) represents a potential therapeutic avenue, though it requires precise regulation to avoid impairing its antioxidant functions.

#### 5.2.3. The Effect of Co on Pancreatitis

As noted in Section 4.4, Co accumulation exacerbates the inflammatory response and fibrotic remodeling in CP by stabilizing HIF-1α under normoxic conditions, which increases the expression of pro-inflammatory mediators [119]. Co also contributes to acinar cell damage by inducing oxidative stress [247].

Conversely, Co deficiency—manifested clinically as vitamin B12 deficiency due to exocrine malabsorption in CP patients—may indirectly impair cellular metabolism and the regenerative capacity of the pancreas [119,248].

However, it must be emphasized that the pathological role of Co accumulation remains primarily based on experimental models. Further robust clinical and epidemiological studies are necessary to clarify its direct pathological impact on human pancreatitis and distinguish it from the well-established nutritional consequences of its deficiency [119,247].

#### 5.2.4. The Effect of I on Pancreatitis

Direct clinical evidence linking nutritional I to pancreatitis is exceptionally limited, and its pathological role remains speculative. In the clinical setting, AP has only been described in isolated case reports as an iatrogenic complication of radioactive I-131 therapy for thyroid disorders. This is likely due to the systemic distribution of the isotope and its toxic accumulation in extra-thyroidal tissues expressing I transporters [249].

Regarding non-radioactive I, experimental animal models suggest that excessive I intake (e.g., Lugol’s solution or potassium iodate) may induce pancreatic oxidative stress, elevate α-amylase activity, and disrupt cellular metabolism by activating transcription factors such as PPAR-γ and CCAAT/enhancer-binding protein beta (C/EBP-β). However, as noted for other elements with purely preclinical data, these experimental findings have not been validated in humans, precluding any definitive clinical conclusions regarding I and pancreatic damage [250].

#### 5.2.5. The Effect of Mn on Pancreatitis

As extensively discussed in Section 4.2, Mn is a critical component of the pancreatic antioxidant defense system, functioning as the essential cofactor for Mn-SOD to prevent oxidative stress [33,103,251].

In the clinical setting of AP, serum Mn-SOD levels serve as a measurable indicator of systemic redox imbalance. Patients with severe AP exhibit significantly elevated serum Mn-SOD concentrations (particularly on days 2 and 5) compared to those with mild AP. These elevated levels strongly correlate with peripheral markers of lipid peroxidation (malondialdehyde), neutrophil activation (myeloperoxidase), and the extent of ischaemia–reperfusion injury, making serial measurements of Mn-SOD a potentially useful clinical marker of treatment efficacy [252].

Conversely, Mn deficiency is clinically relevant in the context of CP, where affected patients exhibit significantly lower dietary Mn intake than healthy controls [253]. Preclinical models indicate that this deficiency profoundly disrupts the synthesis, stability, and storage of pancreatic exocrine enzymes, including amylase. Consequently, Mn dyshomeostasis not only exacerbates oxidative acinar damage but also directly impairs the exocrine secretory function characteristic of CP [103,254].

#### 5.2.6. The Effect of Zn on Pancreatitis

As highlighted in Section 4.2 and Section 4.4, Zn plays a crucial role in maintaining antioxidant defenses and barrier integrity. In AP, preclinical rat models demonstrate that Zn supplementation exerts a potent protective effect via the enteropancreatic axis, reducing endotoxemia, preventing bacterial translocation, and modulating gut microbiota [255]. However, clinical data in AP remain inconsistent [113,256]. While some studies report altered Zn-related redox biomarkers—such as a higher Cu/Zn ratio and lower Zn levels, particularly in smokers—others find no significant differences in Cu/Zn-SOD activity compared to healthy controls, suggesting that Zn disturbances may depend heavily on disease severity and smoking status [113,256].

In CP, clinical evidence strongly positions Zn deficiency as a reliable marker of disease severity and malabsorption [89,90,91,92]. Zn deficiency affects approximately 26% of CP patients and is frequently exacerbated by smoking and advanced age [89,90]. Notably, low serum Zn levels are independently associated with small intestinal bacterial overgrowth (SIBO), highlighting a bidirectional link between Zn status and barrier dysbiosis [89,255]. Furthermore, Zn deficiency has been identified as a clinical risk factor for musculoskeletal complications, such as sarcopenia and osteosarcopenia [91].

Zn-related composite indices, particularly the erythrocyte Zn/Cu ratio, have been proposed as functional biomarkers for exocrine pancreatic insufficiency (EPI), correlating positively with fecal elastase-1 [92]. However, the direct association between absolute Zn levels and EPI status or secondary diabetes remains debated across different cohorts [89,90,91,92]. In AIP, Zn deficiency is present in about 25% of patients and co-occurs with elevated IgG4 levels, suggesting it reflects the broader fibro-inflammatory context of the disease rather than specific clinical subtypes [257].

#### 5.2.7. The Effect of Ag on Pancreatitis

Direct evidence linking Ag to pancreatitis is extremely limited and derives solely from in vivo toxicology studies of AgNPs rather than traditional clinical or experimental pancreatitis models. The reported pancreatic effects are highly contradictory and appear to depend heavily on the formulation, bioavailability, and dose of the nanoparticles [38,258].

On one hand, acute and sub-chronic toxicity studies in Wistar rats demonstrate that while AgNPs cause organ particle deposition, congestion, and injury in major organs like the liver, pancreatic tissue remains unaffected even at higher doses [258]. Conversely, an in silico-guided analysis combined with in vitro and murine toxicity data revealed a pancreatic signal: lethal doses of AgNPs could induce a dose-dependent structural collapse of the pancreas, accompanied by significant elevations in serum amylase and a rise in serum glucose up to approximately 50% [38]. At lethal doses, the pancreas was described as completely “whitened,” which was interpreted as consistent with acute hemorrhagic pancreatitis, and an additional dark, diffuse plaque-like deposit was observed within the damaged pancreatic tissue [38].

Given that these severe manifestations are observed only at lethal toxicity thresholds in murine models, these findings must be interpreted with extreme caution and require confirmation in more reliable models. There is currently no clinical evidence to suggest that Ag exposure plays a pathophysiological role in human acute or chronic pancreatitis [38,258].

#### 5.2.8. The Effect of Cd on Pancreatitis

As extensively detailed in Section 4.2 and Section 4.4, Cd provokes severe oxidative and nitrosative stress, activates canonical inflammatory pathways (e.g., NF-κB), and disrupts ductal physiology [39,112,114,115,116].

In preclinical rat and avian models, acute Cd exposure consistently produces a “pancreatitis-like” biochemical profile characterized by elevated serum amylase and lipase, prominent autophagy, and severe structural disorganization of the exocrine parenchyma [112,114,115,116,259,260]. Importantly, these exocrine injuries are inextricably coupled with profound endocrine disruption; animal models exhibit islet vacuolation and decreased GLUT-2/GLUT-4 expression, leading to dysregulated glucose–insulin homeostasis during pancreatic injury [112,115,116].

Clinically, however, the association between Cd and pancreatitis is primarily tied to tobacco smoking. Studies analyzing human pancreatic tissue and serum confirm that Cd concentrations are significantly higher in smoking patients with CP AP compared to non-smokers [39,113]. As noted in Section 4.4, ex vivo models demonstrate that at tissue concentrations similar to those in human smokers, Cd directly impairs CFTR apical expression, thereby critically inhibiting ductal fluid and bicarbonate secretion [39].

Therefore, while preclinical models illustrate Cd’s capacity to acutely induce an AP-like phenotype via redox imbalance, human data primarily support its role as a chronic, smoking-related toxicant that drives ductal dysfunction and CP pathogenesis [39,112,114,115,116].

#### 5.2.9. The Effect of Hg on Pancreatitis

As comprehensively discussed in Section 4.4, heavy metals like Hg—a prominent toxic component of cigarette smoke—can acutely disrupt exocrine duct physiology [39]. Clinical evaluations by Pallagi et al. [39] demonstrate that peripheral blood Hg concentrations are significantly higher in smokers, including those with CP, compared to non-smokers. Using ex vivo ductal models at concentrations mirroring human smoker serum levels, the same study confirmed that Hg directly impairs apical CFTR localization, thereby severely reducing ductal bicarbonate (HCO_3_^−^) and fluid secretion. This loss of secretory capacity constitutes a key step in the pathogenesis of CP [39].

The role of Hg in driving subsequent pancreatic tissue remodeling is further supported by preclinical in vivo models. In avian models, Hg exposure induces oxidative stress and extensive pancreatic fibrosis [261]. Complementary toxicopathology studies in teleost fish demonstrate direct inorganic Hg deposition within pancreatic acini, leading to pronounced acinar atrophy [262].

Collectively, while the available clinical and experimental evidence strongly connects smoking-related Hg exposure to exocrine pancreatic injury and CP progression via ductal CFTR impairment and profibrotic remodeling, its specific role in AP and AIP remains largely unknown and requires dedicated investigation [39,261,262].

#### 5.2.10. The Effect of Pb on Pancreatitis

As detailed in Section 4.1 and Section 4.2, Pb is a potent inducer of oxidative stress and endocrine dysregulation. However, evidence connecting Pb specifically to pancreatitis is currently limited to experimental in vivo models [67].

In experimental models of Pb acetate exposure, Pb induces profound pancreatotoxicity driven by intracellular ROS. Histopathological evaluations reveal severe degenerative and necrotic changes in the exocrine acinar architecture, accompanied by the shrinkage and atrophy of the islets of Langerhans. This combined structural damage translates to significant endocrine dysfunction, characterized by decreased serum insulin and elevated blood glucose levels [67].

While these experimental findings biologically validate that acute Pb exposure can cause toxic pancreatic tissue damage and metabolic disruption, there is a distinct lack of clinical data confirming that Pb acts as a primary etiologic factor or directly predisposes humans to acute or chronic pancreatitis. Consequently, further translational studies are required to establish its clinical relevance [67].

#### 5.2.11. The Effect of Se on Pancreatitis

As thoroughly established in Section 4.2, Se is a cornerstone of the pancreatic antioxidant defense system. However, in the context of pancreatitis, current evidence relies almost exclusively on experimental in vivo and in vitro models, lacking direct clinical validation [263,264,265,266,267,268].

In rodent models of AP, Se supplementation—administered as sodium salts, quantum dots, or modern nanoparticles—consistently mitigates exocrine injury [263,264,265,266,267,268]. Prophylactic and therapeutic Se administration significantly reduces serum lipase and amylase levels, decreases pancreatic edema, and limits immune cell infiltration [264,265,266,267,268]. Mechanistically, Se strongly activates the Nrf2/Heme oxygenase-1 (HO-1) cytoprotective pathway and suppresses canonical inflammatory cytokines (IL-1β, IL-6, and TNF-α), thereby preventing both primary acinar “self-digestion” and secondary multiorgan damage (e.g., in the lungs and kidneys) [263,264,265,266,267,268]. In specific models of biliary obstruction, Se interventions also effectively reduced bilirubin levels [267].

Importantly, Se also exerts a protective effect on the endocrine compartment during AP episodes. Dose-dependent nanoparticle administration in these models helped preserve the architecture of the islets of Langerhans and partially restored β-cell insulin secretion [265,266]. Furthermore, Se functions as a potent antagonist against heavy metal toxicity. In models of Cd- and Hg-induced pancreatic injury, Se supplementation prevented structural degradation and profibrotic remodeling, acting as an effective cellular antidote [260,261].

While these experimental data highlight Se’s robust potential to silence inflammatory signals and protect both exocrine and endocrine pancreatic functions, comprehensive clinical trials are strictly required to confirm its therapeutic efficacy and safety in human AP and CP.

A summary of the role of trace elements in pancreatitis is given in Table 2.

**Table 2:** The effect of trace elements on pancreatitis. The use of “→ “ denotes causality in the mechanism of the element’s influence on the disease, a down arrow indicates a decrease and an up arrow indicates an increase.

Trace Element	Effect on the Disease	Mechanism of Influence	Additional Information
Iron (Fe) [50,76,97,98,99,100,101,238,239,240]	Negative	**AP:** ↑ ROS (Fenton reaction) → triggers ferroptosis and DAMPs release. **CP:** Fe accumulation → chronic inflammation and fibrotic remodeling.	-
Copper (Cu) [92,102,103,241,242,243,244,245,246]	Negative (in excess)	**AP:** ↓ GPx4 → promotes ferroptosis. **CP:** Induces cuproptosis; shifts redox balance → pro-oxidative states.	However, Cu is also essential in small amounts because it supports antioxidant enzymes SOD1, so its effect depends on balance.
Cobalt (Co) [119,247,248]	Mixed	**CP:** Excess stabilizes HIF-1α → ↑ inflammation & fibrosis. Deficiency (as vitamin B12) impairs regeneration.	-
Iodine (I) [249,250]	Negative	**AP:** Radioactive I-131 → direct radiotoxicity. **CP:** Non-radioactive excess → ↑ oxidative stress; impairment of β -cell function and tissue integrity mediated by altered gene regulation (PPAR- γ and C/EBP- β).	Overall, the effect is negative but very rare and based mainly on isolated case reports.
Manganese (Mn) [33,103,251,252,253,254]	Positive	**AP:** Cofactor for Mn-SOD → ↓ oxidative stress. **CP:** Deficiency → ↓ exocrine enzyme synthesis.	-
Zinc (Zn) [89,90,91,92,113,255,256,257]	Positive	**AP:** Antioxidant cofactor → maintains intestinal barrier. **CP:** Deficiency → reflects severe malabsorption. **AIP:** Deficiency → correlates with IgG4 and fibro-inflammatory process. fibro-inflammatory process.	**In CP**: Zn/Cu-based indices, particularly the erythrocyte Zn/Cu ratio, may reflect exocrine dysfunction, although the association between absolute Zn levels and EPI remains inconsistent across studies.
Silver (Ag) [38,258]	Negative	**AP:** AgNPs → ↑ ROS → dose-dependent pancreatotoxicity and structural acinar collapse.	-
Cadmium (Cd) [39,112,113,114,115,116,259,260]	Negative	**AP:** ↑ NF-κB → severe oxidative/nitrosative stress. **CP:** ↓ CFTR-mediated ductal fluid & HCO_3_^−^ secretion.	-
Mercury (Hg) [39,261,262]	Negative	**CP:** ↓ apical CFTR localization → ↓ ductal secretion → profibrotic remodeling.	Hg levels were higher in the non-smoking CP group than in the non-smoking non-CP group, although the significance of this difference is unclear.
Lead (Pb) [67]	Negative	**AP/CP:** ↑ ROS → pancreatotoxicity → degenerative acinar changes and endocrine dysfunction.	This evidence remains limited to experimental data.
Selenium (Se) [33,92,103,247,248,249,250,251,263,264,265,266,267,268]	Positive	**AP:** ↑ Nrf2/HO-1 pathway → ↓ inflammatory cytokines (IL-1β, IL-6), TNF-α → limits secondary multi-organ damage.	-

Abbreviations: AgNPs—Silver nanoparticles; AIP—Autoimmune pancreatitis; AP—Acute pancreatitis; Cd—Cadmium; CFTR—Cystic fibrosis transmembrane conductance regulator; Co—Cobalt; CP—Chronic pancreatitis; Cu—Copper; Fe—Iron; HCO_3_^−^—Bicarbonate ion; Hg—Mercury; HO-1—Heme oxygenase-1; I—Iodine; IgG4—Immunoglobulin G subclass 4; IL-1β—Interleukin-1 beta; IL-6—Interleukin-6; Mn—Manganese; NF-κB—Nuclear factor kappa B; Nrf2—Nuclear factor erythroid 2–related factor 2; Pb—Lead; PPAR-γ—Peroxisome proliferator-activated receptor gamma; ROS—Reactive oxygen species; Se—Selenium; TNF-α—Tumor necrosis factor alpha; Zn—Zinc; β-cells—Pancreatic beta cell.

### 5.3. The Effect of Trace Elements on Pancreatic Cancer

The last pancreatic disease discussed that is affected by trace elements (see Table 3), PC, predominantly represented by pancreatic ductal adenocarcinoma (PDAC), remains one of the most lethal malignancies worldwide, with a five-year survival rate of less than 10%. Despite advances in oncologic diagnostics and multimodal treatment strategies, the prognosis of PDAC has improved only marginally over the past few decades. This is largely attributable to late-stage diagnosis, aggressive tumor biology, and limited responsiveness to currently available therapies [269].

Clinically, PDAC develops insidiously and is often asymptomatic in its early stages or presents with nonspecific manifestations. Common symptoms at diagnosis include unintentional weight loss, abdominal and back pain, obstructive jaundice, fatigue, steatorrhea, and new-onset DM in adults. As a result, the majority of patients are diagnosed with locally advanced or metastatic disease, which significantly limits curative treatment possibilities [269].

Established risk factors for PC include advanced age, tobacco smoking, obesity, T2DM, CP, and inherited genetic susceptibility. Germline mutations in genes such as *BRCA1*, *BRCA2*, *PALB2*, *CDKN2A*, and *ATM* have been associated with increased PDAC risk, underscoring the importance of genetic and metabolic predisposition in disease development [270].

Given the complexity of PDAC biology and persistently dismal clinical outcomes, there is an urgent need to improve understanding of the molecular, metabolic, and microenvironmental factors that influence PC initiation, progression, and therapeutic response. Systematic evaluation of emerging mechanistic and translational evidence is essential for identifying novel biomarkers and therapeutic targets that may enable earlier diagnosis and improve survival outcomes for patients with PC [271].

#### 5.3.1. The Effect of Fe on Pancreatic Cancer

As detailed in Section 4.2 and Section 4.3, profound dysregulation of Fe metabolism is a central molecular hallmark of PDAC. PDAC cells actively remodel Fe trafficking pathways to maximize intracellular Fe availability while simultaneously evading Fe-induced ferroptotic cell death [51,52,53,104,105,106].

Mechanistically, this is achieved through sustained upregulation of the Fe import machinery (predominantly the transferrin receptor, TfR) and concurrent suppression of Fe export via the FPN1 axis [52,54,55,56,57]. This coordinated dysregulation effectively locks Fe within tumor cells and tumor-associated macrophages (TAMs) [52,56,57].

As previously outlined (Section 4.2), this expanded labile Fe pool synergizes with oncogenic KRAS signaling, driving Fenton-mediated genomic instability and activating survival pathways such as MAPK and PI3K–Akt [51,55,78,79,80]. Beyond redox signaling, intracellular Fe accumulation is indispensable for tumor metabolic reprogramming. It acts as a critical cofactor for ribonucleotide reductase (essential for rapid DNA synthesis and S-phase progression) and sustains mitochondrial oxidative phosphorylation via Fe-S clusters and heme moieties, even within the hypoxic and nutrient-restricted PDAC stroma [53,272,273,274].

Furthermore, as discussed in Section 4.4, Fe-driven oxidative stress profoundly shapes the non-malignant components of the tumor microenvironment. It activates pancreatic stellate cells, driving the dense desmoplasia characteristic of PDAC, and polarizes TAMs toward an immunosuppressive, pro-tumorigenic state [56,120,121,275,276].

At the systemic level, this tumor-driven chronic activation of the hepcidin pathway leads to macrophage Fe sequestration and reduced dietary absorption. Clinically, this manifests as functional Fe deficiency and the anemia of chronic disease, which severely exacerbates fatigue and cachexia, ultimately reducing patient tolerance to aggressive anticancer therapies [57,277].

#### 5.3.2. The Effect of Cu on Pancreatic Cancer

As delineated in Section 4.2 and Section 4.3, Cu homeostasis is actively rewired in PDAC to support malignant growth while avoiding Cu-induced cytotoxicity (cuproptosis) [32,109,110,248].

At the cellular level, PDAC cells exhibit significantly enhanced Cu uptake, primarily driven by the robust upregulation of the high-affinity Cu transporter SLC31A1 (CTR1) [110,111]. As previously discussed (Section 4.2), this elevated intracellular Cu pool is exploited by the tumor to drive moderate oxidative stress, acting as a signaling mechanism to promote oncogenic pathways (e.g., MAPK and PI3K–Akt) [51,54]. Concurrently, this Cu influx is indispensable for maintaining PDAC bioenergetics; experimental silencing or pharmacological inhibition of CTR1 critically disrupts mitochondrial respiration and ATP production. To survive this Cu burden, cancer cells rely on robust adaptive systems, utilizing autophagy and antioxidant defenses to buffer oxidative damage and limit cuproptotic vulnerability [110,111].

Beyond the malignant epithelial cells, Cu dysregulation profoundly shapes the tumor microenvironment. Elevated Cu levels are critical for angiogenesis, directly supporting endothelial cell migration and activating pro-angiogenic mediators such as vascular endothelial growth factor (VEGF), thereby facilitating tumor expansion and early metastasis [278,279].

Clinically, metallomic profiling reveals distinct Cu signatures in PDAC tissues compared to non-malignant pancreas, and dynamic changes in serum Cu concentrations during anticancer therapy suggest its potential utility as a biomarker of treatment response [248,280]. Therapeutically, exploiting this altered Cu handling represents a highly promising strategy. Preclinical models demonstrate that pharmacological interference with Cu efflux pumps (ATP7A and ATP7B) sensitizes PDAC cells to platinum-based chemotherapy (e.g., cisplatin) by enhancing intracellular drug accumulation [281,282]. Alternatively, using synthetic Cu complexes or Cu ionophores to intentionally exceed the tumor’s buffering capacity results in massive mitochondrial ROS accumulation, effectively triggering intrinsic apoptotic pathways and cuproptosis [281,282,283,284].

#### 5.3.3. The Effect of Co on Pancreatic Cancer

Retrospective cohort and metallomic profiling studies indicate that systemic Co homeostasis is altered in PDAC, with significantly elevated serum Co levels observed at diagnosis compared to healthy controls [109,248,280]. However, unlike Zn or Se, clinical data demonstrate that Co concentrations do not consistently correlate with tumor stage, metastatic burden, or overall survival [280]. Therefore, Co currently appears to function merely as a contextual biomarker reflecting systemic metabolic and inflammatory alterations, rather than a primary, independent prognostic driver of disease progression [280].

Direct mechanistic studies evaluating Co specifically in PDAC models are currently lacking. As discussed in Section 4.4, Co is a recognized chemical mimetic of hypoxia that stabilizes HIF-1α, potentially reinforcing the hypoxia-driven transcriptional programs (e.g., angiogenesis and stromal activation) that characterize the dense PDAC microenvironment [56,285]. Additionally, Co may contribute to ROS-mediated genomic instability and oncogenic KRAS signalling, as detailed in Section 4.2 [51,55]. Moreover, identified correlations between Co concentrations and inflammatory markers suggest it may indirectly influence stromal activation and immune cell function within this microenvironment. Nevertheless, these biologically plausible roles remain purely inferential in the context of pancreatic malignancy, highlighting the absolute necessity for targeted experimental and clinical studies to define Co’s functional contribution to PDAC [109,273,280,286].

#### 5.3.4. The Effect of I on Pancreatic Cancer

Despite extensive research into trace elements, I has not been identified as a biological mediator of PDAC pathogenesis [32,51,54,55]. No experimental in vitro or in vivo studies demonstrate an effect of I on pancreatic cancer cell proliferation, apoptosis, invasion, or metastatic potential, nor do epidemiological investigations support an association between dietary I intake and disease incidence [32,51,54,55]. In stark contrast to elements like Fe or Cu, clinical and experimental consensus indicates that I is not a molecular regulator of pancreatic cancer biology [109,248,280].

The established relevance of I in PDAC is strictly confined to clinical diagnostics and targeted radiotherapy. Iodinated contrast agents are indispensable for contrast-enhanced computed tomography (CT) to facilitate tumor detection, staging, and quantitative I mapping [248,273,286]. Therapeutically, I is utilized exclusively as a radioisotope (e.g., I-125) for interstitial brachytherapy in locally advanced or unresectable PDAC [54,56]. However, the antitumor efficacy of I-125 is derived entirely from localized ionizing radiation, inducing DNA damage, rather than any intrinsic biochemical or metabolic activity of the I element itself [54,56].

#### 5.3.5. The Effect of Mn on Pancreatic Cancer

As detailed in Section 4.2, Mn homeostasis is a critical regulator of mitochondrial redox balance via Mn-SOD [51,55,109]. Current evidence does not support Mn as a direct carcinogen in PDAC. Instead, reduced expression or activity of Mn-SOD is frequently observed in PC tissues and cell lines, contributing to the mitochondrial ROS accumulation and genomic instability (synergizing with oncogenic KRAS) discussed in Section 4.2 [32,51,55,87]. Experimental restoration of Mn-SOD effectively suppresses tumor growth, confirming its inherent tumor-suppressive role in the pancreas [87,287].

Clinically, altered systemic Mn distribution is associated with disease stage and poorer survival outcomes, suggesting it primarily acts as a biomarker reflecting an aggressive metabolic phenotype rather than a direct driver of disease progression [248,273]. Within the tumor microenvironment (as outlined in Section 4.4), excess ROS resulting from Mn-SOD dysfunction may contribute to stromal fibroblast activation and desmoplasia; however, direct in vivo clinical evidence confirming Mn’s specific role in PDAC immune suppression remains scarce [56,109,121,288,289,290,291,292].

Therapeutically, Mn is not a conventional targeted pathway. Rather, Mn-based nanomaterials and redox-active compounds (e.g., manganoporphyrins) are being experimentally leveraged to exploit the redox vulnerability of PDAC cells. In preclinical models, these compounds successfully induce lethal oxidative stress, promote ferroptosis, and enhance radiosensitivity [289,290,291,293,294]. Nevertheless, these promising strategies remain strictly experimental and require significant translational validation before clinical application.

#### 5.3.6. The Effect of Zn on Pancreatic Cancer

Clinical and epidemiological evidence strongly implicates Zn in the pathogenesis of PDAC. Meta-analyses demonstrate that higher dietary Zn intake is associated with a significantly reduced risk of developing PDAC [295]. Conversely, in clinical settings, systemic Zn deficiency is highly prevalent among patients with inoperable PDAC and serves as an independent prognostic factor for poor overall survival [296].

At the tissue level, the role of Zn is highly dynamic and dictated by a profound “transporter paradox” during disease progression. During early carcinogenesis, such as the progression of pancreatic intraepithelial neoplasia (PanIN), exocrine cells markedly downregulate the RREB1/ZIP3 axis. This deliberately limits intracellular Zn accumulation, allowing early cancer cells to evade Zn-induced cytotoxicity [297,298,299]. In stark contrast, advanced PDAC tumors paradoxically overexpress the ZIP4 transporter. This ZIP4-mediated Zn influx drives aggressive proliferation, epithelial–mesenchymal transition (EMT), and metastasis by activating key oncogenic transcription factors, including Signal transducer and activator of transcription 3 (STAT3) and ZEB1 [297,300,301,302]. High expression of ZIP4, alongside other transporters (ZIP11, ZnT1, ZnT6) and zinc finger proteins (ZNF655, ZNF488), strongly correlates with poor patient prognosis, positioning these molecules as potential biological markers [303,304,305,306].

Because PDAC cells depend on a narrow, tightly controlled window of intracellular Zn, they exhibit a unique therapeutic vulnerability to its dysregulation. Preclinical studies demonstrate that both acute Zn deprivation (using membrane-permeable chelators like TPEN) and targeted Zn overload (via exogenous Zn or ZnS-PVP nanoparticles) induce lethal oxidative stress, mitochondrial depolarization, and autophagic blockade [307,308,309]. While these targeted nanomedical approaches have shown remarkable efficacy in overcoming chemoresistance in mouse models, they remain strictly experimental and await comprehensive clinical validation.

#### 5.3.7. The Effect of Ag on Pancreatic Cancer

Unlike trace elements integrated into human metabolism, the role of Ag in PDAC is currently confined strictly to the realm of experimental nanomedicine and targeted therapeutics, with no epidemiological data linking it to disease pathogenesis [310,311].

Preclinical in vitro and in vivo (xenograft) models demonstrate that Ag, both in its ionic form (e.g., silver nitrate complexes) and as AgNPs, exerts potent, dose- and size-dependent cytotoxicity against PDAC cells [310,312]. Mechanistically, AgNPs eradicate pancreatic cancer cells by inducing massive intracellular ROS generation, leading to mitochondrial damage and triggering multiple forms of cell death, including apoptosis, necroptosis, and lethal autophagy [310,311,313,314]. At the molecular level, this cytotoxicity involves the activation of the MAPK pathway and altered expression of key regulatory proteins, including the upregulation of Bax, p53, RIP1/3, and MLKL, along with the downregulation of Bcl-2 [310,311]. Furthermore, experimental AgNP administration strongly inhibits critical oncogenic behaviors, including cell migration, colony formation, and spheroid growth [311].

However, the primary barrier to the clinical translation of Ag-based therapeutics is their unpredictable nanotoxicity. While several studies report that AgNPs selectively target cancer cells over non-malignant models, other investigations reveal significant off-target toxicity to healthy human cells, which can sometimes exceed the antineoplastic effects [310,314,315,316]. This therapeutic window is strictly dependent on the nanoparticles’ size, synthesis method, and specific surface coatings [315,316].

Consequently, realizing the potential of Ag in PDAC therapy requires rigorous physicochemical optimization. Furthermore, concurrent protective strategies, such as the co-administration of antioxidants, such as α -lipoic acid, have been experimentally shown to mitigate systemic and hepatic nanotoxicity without compromising the antineoplastic efficacy of AgNPs against PDAC, highlighting a critical pathway for future translational research [317].

#### 5.3.8. The Effect of Cd on Pancreatic Cancer

Unlike elements studied primarily for experimental therapeutics, Cd is a well-established environmental risk factor for PC development.

Robust clinical evidence, including a 2025 systematic review and meta-analysis, demonstrates that individuals with high Cd exposure have approximately twice the risk of developing PC compared with those with low or no exposure [318]. Crucially, this relationship exhibits a positive dose–response pattern, where incrementally higher levels of Cd biomarkers correlate with a progressively escalating cancer risk [319]. Large prospective cohort studies further corroborate these findings, linking elevated urinary Cd concentrations—a reliable biomarker of chronic, long-term exposure—to significantly increased PC-specific mortality [320].

Elevated whole-blood and serum Cd concentrations are consistently observed in PC patients across diverse geographical cohorts [321,322]. Importantly, while tobacco smoke is a primary source of Cd, prospective case–control studies confirm that the carcinogenic risk remains statistically significant even after adjusting for smoking status. This highlights that chronic dietary and environmental Cd exposure (e.g., via polluted soil and water in regions such as the Nile Delta) independently drives pancreatic carcinogenesis [321,322].

At the cellular level, the mechanisms underlying Cd-induced malignancy are profound. Long-term exposure to low-dose Cd actively transforms normal pancreatic duct epithelial cells into highly aggressive phenotypes. This malignant transformation is characterized by increased cellular invasion, the secretion of matrix metalloproteinase (MMP) 9, and the pronounced overexpression of the PC marker S100P. Furthermore, chronic Cd exposure promotes the formation of “pancreaspheres”—populations of cells exhibiting cancer stem cell (CSC)-like properties. These Cd-induced CSCs demonstrate heightened resistance to apoptosis, form aggressive, poorly differentiated gland-like structures in 3D cultures, and drive tumor initiation and progression, thereby solidifying the critical role of Cd in the pancreatic cancer etiology [323].

#### 5.3.9. The Effect of Hg on Pancreatic Cancer

Unlike Cd, the epidemiological and clinical evidence linking Hg to PC is currently limited and somewhat paradoxical [248,324].

Recent clinical evaluations have revealed complex distribution patterns of Hg in affected patients. A 2024 study demonstrated that serum Hg levels were significantly lower in PC patients than in healthy controls [248]. However, at the tissue level, Hg concentration in non-malignant pancreatic parenchyma appears to correlate with malignant progression; Hg levels were higher in healthy tissue adjacent to overt PDAC than in tissue adjacent to precancerous intraductal papillary mucinous neoplasms (IPMN) [248]. Supporting the concept of localized tissue accumulation, earlier histological analyses revealed that inorganic Hg is significantly more prevalent in the functional pancreatic compartments—including islet, acinar, and ductal cells—of PC patients than in healthy individuals [324].

While these preliminary findings suggest that localized Hg accumulation might be associated with pancreatic carcinogenesis, the data are currently too sparse and contradictory to establish Hg as a definitive environmental risk factor or a reliable diagnostic tumor biomarker [248,324]. Comprehensive, large-scale studies are strictly required to determine whether Hg deposition acts as a biological driver of malignant transformation or merely represents an epiphenomenon of the altered tissue microenvironment in PDAC.

#### 5.3.10. The Effect of Pb on Pancreatic Cancer

Unlike other heavy metals, research specifically isolating the effect of Pb on PC remains exceptionally sparse. Recent clinical evaluations from 2024 highlight significant, yet complex, alterations in Pb distribution. Systemically, serum Pb levels are significantly elevated in patients with PDAC compared to healthy individuals [248].

However, this systemic elevation contrasts with localized tissue dynamics. Pb concentrations within the actual tumor tissue are unexpectedly lower than in the adjacent non-malignant parenchyma. Furthermore, mirroring the specific progression patterns observed with Hg, Pb levels in overt PDAC tissue are higher than those found in IPMN tissue [248].

While these spatial concentration gradients suggest that localized Pb mobilization and systemic redistribution may accompany malignant transformation, the current lack of corroborating mechanistic studies or large-scale epidemiological data precludes any definitive conclusions regarding Pb as a causal, biological driver in PDAC pathogenesis [248].

#### 5.3.11. The Effect of Se on Pancreatic Cancer

Clinical and epidemiological data robustly position Se as a significant factor in both PDAC risk and prognosis. Meta-analyses consistently demonstrate that higher dietary Se intake is associated with a statistically significant reduction in the risk of developing PC [325,326]. However, clinical evidence introduces a critical caveat: this protective benefit is derived primarily from a natural diet. Se supplementation in non-deficient populations lacks a clear prophylactic effect and may paradoxically negate the protective benefits of dietary Se [326].

In patients with established PDAC, serum Se levels are significantly reduced, and this deficiency is a strong independent prognostic factor for shorter overall survival [248]. Furthermore, a 2024 study monitoring trace elements during active PC treatment revealed that a progressive decline in serum Se levels during therapy is strongly correlated with an increased risk of mortality, underscoring its potential utility as a dynamic biomarker of treatment response and disease progression [280].

Mechanistically, as detailed in Section 4.2, Se exerts its prophylactic effects primarily by incorporating into GPx1, neutralizing hydrogen peroxide and protecting pancreatic DNA from oncogenic damage [327].

In the therapeutic realm, Se is being actively investigated in preclinical models. While certain Se compounds can induce targeted cell death via ferroptosis [32], the most promising experimental avenue involves combinatorial therapies. In vitro studies show that adding Se compounds to gemcitabine—the standard-of-care chemotherapeutic for PDAC—synergistically enhances cancer cell apoptosis [328]. Additionally, emerging nanomedical approaches utilizing SeNPs have demonstrated efficacy in inhibiting PDAC proliferation by blocking the mTOR pathway and inducing autophagy [329]. However, these promising Se-based therapeutic interventions remain strictly in the preclinical phase and await rigorous clinical validation.

A summary of the role of trace elements in pancreatitis is given in Table 3.

**Table 3:** The effect of trace elements on pancreatic cancer. The use of “→ “ denotes causality in the mechanism of the element’s influence on the disease.

Trace Element	Effect on the Disease	Mechanism of Influence	Additional Information
Iron (Fe) [51,52,53,54,55,56,57,78,79,80,104,105,106,120,121,272,273,274,275,276,277]	Negative	Fe overload → excessive ROS via Fenton reaction → oxidative stress → DNA damage and ferroptosis	Promotes mutations and carcinogenesis; high Fe levels are linked to increased PC risk
Copper (Cu) [32,51,54,109,110,111,248,278,279,280,281,282,283,284]	Mixed (mainly negative)	Excess Cu → ROS generation via Fenton-like reactions → mitochondrial dysfunction and cellular damage	Contributes to chronic inflammation; involvement in cuproptosis pathways in cancer cells
Cobalt (Co) [51,55,56,109,248,273,280,285,286]	Mixed (mainly negative)	High levels → ROS production → disrupts Ca^2+^ signaling and inhibits insulin secretion	Toxic in excess; affects cellular metabolism and may promote a pro-tumorigenic environment
Iodine (I) [32,51,54,55,56,109,248,273,280,286]	Does not affect Pc development	-	Its relevance in PDAC is limited in diagnostic imaging and selected radiotherapeutic applications
Manganese (Mn) [32,51,55,56,87,109,121,248,273,287,288,289,290,291,292,293,294]	Mixed	Cofactor of Mn-SOD → antioxidant defense; imbalance → mitochondrial dysfunction	Both deficiency and excess impair pancreatic function and cellular redox status
Zinc (Zn) [295,296,297,298,299,300,301,302,303,304,305,306,307,308,309]	Positive	Exogenous Zn (at appropriate doses) → selective cytotoxicity to PC cells; induces oxidative stress and autophagy blockade in tumors	PC patients often show systemic Zn deficiency; Zn depletion via TPEN can trigger cancer cell death
Silver (Ag) [310,311,312,313,314,315,316,317]	Positive	AgNPs → induce apoptosis and mitochondrial damage via ROS generation	Potential therapeutic application of AgNPs in targeted PC treatment
Cadmium (Cd) [318,319,320,321,322,323]	Negative	Chronic exposure → malignant transformation of pancreatic duct epithelium	Strong carcinogen; elevated Cd levels correlate with higher PC incidence and mortality
Mercury (Hg) [248,324]	Negative	Accumulation of inorganic Hg in pancreatic tissue → oxidative stress and proteotoxicity	Inorganic Hg is significantly more prevalent in the pancreas of PC patients
Lead (Pb) [248]	Negative	Induction of ROS → DNA damage → interference with DNA repair mechanisms	Elevated Pb levels are frequently observed in patients with PDAC
Selenium (Se) [32,280,325,326,327,328,329]	Positive	Se (via GPx1) → neutralizes H_2_O_2_ → protects DNA from damage; SeNPs inhibit mTOR pathway	Se compounds induce apoptosis in PC cells; serum Se levels are significantly reduced in PDAC patients

Abbreviations: Ag—Silver; AgNP—Silver nanoparticles; Cd—Cadmium; Co—Cobalt; Cu—Copper; Fe—Iron; GPx1—Glutatione peroxidise 1; Hg—Mercury; I—Iodine; Mn—Manganese; Mn-SOD—Manganese superoxide dismutase; Nrf2—Nuclear factor erythroid-2-related factor-2; Pb—Lead; PC—Pancreatic cancer; PPAR-γ—Peroxisome proliferator-activated receptor gamma; ROS—Reactive Oxygen Species; Se—Selenium; SeNPs—Selenium nanoparticles; Zn—Zinc.

## 6. Interactions Between Trace Elements

The diverse pathological effects of trace elements in the pancreas converge on several common mechanistic axes that bridge the gap between DM, pancreatitis, and PC, with the inflammatory response acting as a unifying driver. The primary axis is the redox imbalance, where the depletion of Se and Zn, combined with the catalytic activity of Fe and Cu, creates a pro-oxidant environment. This oxidative stress not only triggers specific cell death pathways but also activates the NF-κB and MAPK/JNK signaling cascades, promoting the release of pro-inflammatory cytokines (TNF-α, IL-6, IL-1β). These cytokines exacerbate tissue damage and further reduce the inherently low antioxidant capacity of β-cells, making them a primary target for Cd and Pb-induced apoptosis. Another critical axis is the programmed cell death network, where Cu and Fe orchestrate cuproptosis and ferroptosis, respectively. While these processes drive acinar cell destruction in AP, the resulting cellular debris further fuels an inflammatory-fibrotic loop, characterized by stellate cell activation and chronic structural remodeling. In the context of malignancy, a secretory–inflammatory axis exists, in which heavy metals such as Hg and Cd disrupt CFTR-mediated bicarbonate secretion; the resulting ductal dysfunction initiates a persistent inflammatory milieu and an acidic microenvironment. This chronic inflammation facilitates oncogenic transformation while cancer cells simultaneously hijack Se-dependent GPx4 systems to evade ferroptosis. These common pathways suggest that various pancreatic pathologies are essentially driven by a self-perpetuating cycle of disturbances in trace element homeostasis and the recruitment of inflammatory cells.

**Figure 4:**
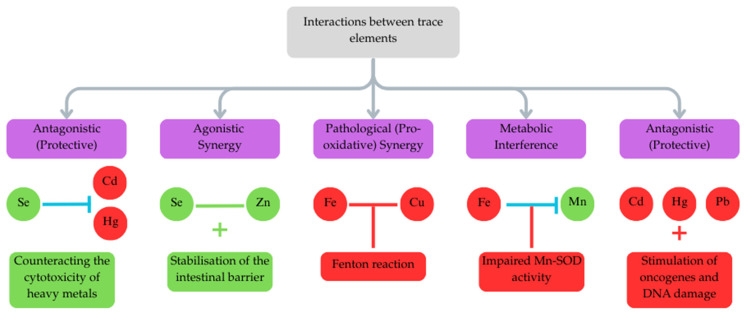
Interactions between trace elements affecting the pancreas. Green lines indicate a beneficial effect on health, red indicates a negative effect, blue indicates inhibition, and a plus sign indicates stimulation of the process Abbreviations: Cd—Cadmium; Cu—Copper; Fe—Iron; Hg—Mercury; Mn—Manganese; Mn-SOD—Manganese superoxide dismutase; Pb—Lead; Se—Selenium; Zn—Zinc.

Trace elements can also interact with one another in agonistic and antagonistic ways, thereby affecting the physiology and function of the pancreas (Figure 4). In antagonistic interactions, the key feature is the antagonism between antioxidant elements and heavy metals. Se plays a priority role here, demonstrating the ability to directly counteract structural damage to the organ caused by Cd and inorganic Hg. On the other hand, Zn and Se exhibit positive agonistic effects, which together stabilise the intestinal barrier, preventing dangerous bacterial translocation in the course of AP. On the other hand, there are also dangerous synergies between elements: Fe and Cu, when present simultaneously at high concentrations, act as catalysts for the Fenton reaction, generating excess ROS, which underlines the pathogenesis of diabetes and inflammation. In addition, excess Fe can disrupt Mn metabolism, reducing Mn-SOD activity. These negative interactions are exacerbated by the accumulation of heavy metals such as Cd, Hg, and Pb, which together promote apoptosis of pancreatic islet β cells and increase insulin resistance. In the course of pancreatic inflammation, Cd and Hg act together to block CFTR channels in the excretory ducts, which drastically reduces bicarbonate secretion and leads to the destruction of the exocrine part of the organ. In the context of cancer, trace elements form a complex network of dependencies: while heavy metals jointly stimulate oncogenes and damage DNA, pancreatic cancer cells use Se-dependent systems (e.g., the GPx4 enzyme) to build resistance to ferroptosis.

## 7. Future Perspectives

While the role of trace elements in pancreatic pathologies is biologically profound, their direct clinical translation remains challenging. It is imperative to clearly distinguish what is currently clinically feasible from what remains purely speculative.

At present, no single elemental biomarker is genuinely ready for standalone clinical use in diagnosing or staging pancreatic diseases. Although circulating markers such as the Cu/Zn ratio or urinary Cd correlate with metabolic dysfunction and disease severity, their low organ specificity limits their utility to an adjunctive role. Furthermore, the clinical implementation of these biomarkers is hindered by unresolved issues regarding sensitivity, specificity, and reproducibility. The lack of standardized analytical protocols, the absence of universally accepted disease-specific reference ranges, and the frequent discrepancy between systemic blood levels and actual intra-pancreatic tissue concentrations severely restrict their diagnostic reliability. To overcome the poor specificity of single elements, developing multi-element metallomic panels represents a logical next step. However, while technologically possible using advanced spectrometry (e.g., ICP-MS), the high cost, complex data interpretation, and lack of standardization currently render such panel analyses impractical for routine clinical screening.

Regarding therapeutic interventions, the evidence base is highly stratified. Based on robust randomized controlled trial evidence, targeted Zn supplementation in patients with T2DM who are deficient in Zn is a feasible and validated strategy to improve glycemic control. Conversely, randomized controlled trial data for Se supplementation yield highly inconsistent results, demonstrating a narrow therapeutic window and a U-shaped risk curve. Crucially, indiscriminate supplementation poses severe toxicity risks; over-supplementation of Se in non-deficient individuals can paradoxically induce insulin resistance, while excess Fe and Cu directly catalyze Fenton reactions, driving ferroptotic and cuproptotic cell death that accelerates pancreatic tissue destruction. Therefore, any nutritional intervention must be strictly guided by personalized deficiency profiling rather than generalized supplementation.

Finally, while novel therapeutic strategies—such as ZIP4 pathway blockade, targeted modulation of ferroptosis/cuproptosis, and the use of Ag or Se NPs show remarkable antineoplastic potential in preclinical PDAC models, they remain entirely speculative. These nanomedical and targeted approaches face immense toxicological and pharmacological hurdles and are years away from clinical feasibility, requiring rigorous validation in future human trials.

## 8. Limitations

This article, which focuses on the role of trace elements in the functioning and pathologies of the pancreas, is a review. Despite the wide range of data discussed, it is not a systematic review, which entails certain methodological limitations. The methodology described above allows for the description of many aspects of the role of individual elements, but the lack of an approach used in systematic reviews limits the assessment of the strength of conclusions from individual studies. There is a lack of large-scale, longitudinal interventional studies. For this reason, it is difficult to establish a clear causal link between disturbances in trace element homeostasis and the development of pancreatic diseases. The information contained in the individual chapters comes from various studies: animal studies, observational studies in humans, and interventional studies, which differ in study design, exposure methods, duration, and measurement methods. Citing results is necessary to show the broader perspective of the problem under study, but individual study results can be inconsistent, underscoring the need for further research.

## 9. Conclusions

A review of the literature confirms that trace elements are a key component of pancreatic homeostasis, regulating both its endocrine and exocrine functions. However, to effectively translate these findings into practice, it is critical to prioritize elements based on their current clinical relevance versus those that remain strictly exploratory.

Elements such as Fe, Cu, Zn, Cd, and Se demonstrate the highest clinical and translational relevance. Zn plays a definitive role in diabetes pathogenesis; its deficiency drastically reduces the pancreatic secretory capacity, making it an actionable biomarker and a validated target for supplementation. Cd is firmly established epidemiologically as a potent environmental carcinogen that correlates strongly with aggressive tumor progression in pancreatic cancer. Fe, Cu, and Se are central to redox balance; excessive accumulation of Fe and Cu drives specific cell death pathways (ferroptosis and cuproptosis) and chronic inflammation common to diabetes and pancreatitis, while severe declines in Se impair antioxidant defenses and promote exocrine tissue destruction.

Conversely, elements such as Ag, Pb, Co, and I currently remain entirely in the exploratory and toxicological domain. While targeted therapeutic modulation—such as the use of Ag or Se nanoparticles—shows remarkable preclinical potential for sensitizing pancreatic cancer cells to chemotherapy, these interventions remain speculative and are far from clinical application.

Crucially, the interpretation of these metallomic findings must be explicitly contextualized within the inherent limitations of the available evidence. Much of the current mechanistic understanding—particularly regarding novel cell death pathways and the efficacy of nanomedicines—relies heavily on preclinical in vitro and animal models. These models often fail to accurately replicate the chronic, low-dose exposure patterns and the complex multi-element interactions characteristic of human pathology. Furthermore, the notable lack of large-scale, longitudinal intervention studies precludes establishing a definitive causal relationship between trace element dyshomeostasis and the onset of pancreatic diseases.

Therefore, while understanding the interactions between trace elements and the pancreatic microenvironment offers an innovative framework for early diagnosis and therapy, future progress requires a shift from isolated, preclinical observations to rigorously validated, prospective clinical trials.

## Data Availability

Not applicable.

## Abbreviations

The following abbreviations are used in this manuscript:

Ag	Silver
AgNPs	Silver nanoparticles
AIP	Autoimmune pancreatitis
Akt	Protein kinase B
AP	Acute pancreatitis
ATP	Adenosine triphosphate
Bcl-2	B-cell lymphoma 2
BMI	Body mass index
BMP	Bone morphogenetic protein
C/EBPα	CCAAT/enhancer-binding protein alpha
C/EBP-β	CCAAT/enhancer-binding protein beta
Cat	Catalase
Cd	Cadmium
CFTR	Cystic fibrosis transmembrane conductance regulator
Co	Cobalt
CoCl_2_	Cobalt chloride
COX-2	Cyclooxygenase-2
CP	Chronic pancreatitis
CTR1 (SLC31A1)	Copper transporter 1
Cu	Copper
Cu/Zn-SOD	Copper/zinc superoxide dismutase
DAMPS	Damage-associated molecular patterns
DM	Diabetes mellitus
EPI	Exocrine pancreatic insufficiency
ERCP	Endoscopic retrograde cholangiopancreatography
Fe	Iron
Fe^2+^	Ferrous iron
Fe^3+^	Ferric iron
Fe–S	Iron–sulfur (cluster)
FPN1	Ferroportin 1
FSH	Follicle-stimulating hormone
FSP1	Ferroptosis suppressor protein 1
GDM	Gestational diabetes mellitus
GLUT	Glucose transporter type
GPx	Glutatione peroxidase
GSH	Glutathione
HbA1c	Glycated hemoglobin
HFE	Hemochromatosis gene
Hg	Mercury
hIAPP	Human islet amyloid polypeptide
HIF-1α	Hypoxia-inducible factor-1 alpha
HJV	Hemojuvelin
HO-1	Heme oxygenase-1
I	Iodine
IgG4	Immunoglobulin G4
IL-6	Interleukin-6
JNK	c-Jun N-terminal kinase
KRAS	Kirsten rat sarcoma viral oncogene homolog
MafA	V-maf musculoskeletal fibrosarcoma oncogene homolog A
MAPK	Mitogen-activated protein kinase
Mcl-1	Myeloid Cell Leukaemia 1
MDA	Malondialdehyde
Mdm-2	Mouse Double Minute 2 homolog
MeHg	Methylmercury
MMP	Matrix metalloproteinase
Mn	Manganese
Mn-SOD	Manganese superoxide dismutase
MODY	Maturity-onset diabetes of the young
MRCP	Magnetic Resonance Cholangiopancreatography
MSRB1	Methionine reductase-R -sulphoxide reductase B1
NF-κB	Nuclear factor kappa B
NQO1	NAD(P)H:quinone oxidoreductase 1
Nrf2	Nuclear factor erythroid-2-related factor-2
NTBI	Non-transferrin-bound iron
Pb	Lead
PDAC	Pancreatic ductal adenocarcinoma
PDX1	Pancreatic and duodenal homeobox 1
PI3K	Phosphoinositide 3-kinase
PI3K/Akt	Phosphatidylinositol 3-kinase/protein kinase Akt
PPARγ	Peroxisome proliferator-activated receptor gamma
RBC	Red blood cell
ROS	Reactive oxygen species
Se	Selenium
SELENOK	Selenoprotein K
SELENOP	Selenoprotein P
SELENOS	Selenoprotein S
SELENOW	Selenoprotein W
SIBO	Small intestinal bacterial overgrowth
siRNA	Small interfering ribonucleic acid
SMAD	Mothers against decapentaplegic
SOD	Superoxide dismutase
STAT3	Signal transducer and activator of transcription 3
T1DM	Type 1 diabetes mellitus
T2DM	Type 2 diabetes mellitus
TAMs	Tumor-associated macrophages
TfR1	Transferrin receptor 1
TfR2	Transferrin receptor 2
TNF-α	Tumor necrosis factor alpha
TrxR	Thioredoxin reductase
VEGF	Vascular endothelial growth factor
ZIP14	Zrt- and Irt-like protein 14
Zn	Zinc
ZnT8	Zinc transporter 8
